# Stance-phase kinematic descriptors differentiate clinical subgroups in women with moderate knee osteoarthritis

**DOI:** 10.3389/fbioe.2026.1903640

**Published:** 2026-08-18

**Authors:** Mayra A. Loayza-Saldaña, Simone Tassani, Laura Tío, Jordi Monfort, Gil Serrancolí, Gemma Piella, Jérôme Noailly

**Affiliations:** 1 Department of Engineering, Universitat Pompeu Fabra, Barcelona, Spain; 2 Inflammation and Cartilage Research Group, Hospital del Mar Research Institute, Barcelona, Spain; 3 Rheumatology Service, Hospital del Mar, Barcelona, Spain; 4 Simulation and Movement Analysis Lab, Universitat Polit `ecnica de Catalunya, Barcelona, Spain; 5 Computational Robotics Group, Institut de Robòtica i Informàtica Industrial (CSIC-UPC), Barcelona, Spain

**Keywords:** gait analysis, knee osteoarthritis, machine learning, patient stratification, stance-phase kinematics

## Abstract

**Introduction:**

Total knee replacement (TKR) is the standard surgical treatment for severely symptomatic knee osteoarthritis (KOA), but treatment decisions for patients with moderate radiographic severity (Kellgren–Lawrence grades 2–3) remain variable. Radiographic grading and patient-reported outcomes do not fully capture the functional heterogeneity between treatment pathways. Stance-phase kinematics may provide objective descriptors for stratification while remaining more accessible than kinetic measurements.

**Methods:**

Sixty-six women with moderate KOA (27 scheduled for TKR, 39 managed conservatively) underwent gait analysis. Stance-phase angles for 12 variables were extracted at three sub-phases (loading response, mid-stance, terminal stance). Group differences were assessed via repeated-measures multifactorial MANOVA. Gait speed was evaluated for confounding (Rothman’s criteria) and included as a covariate in MANCOVA. Stance-phase change variables trained a Random Forest classifier with nested cross-validation and exhaustive feature selection. Associations between descriptors and clinical outcomes were examined via univariate logistic regression. Clinical construct consistency was assessed against the Osteoarthritis Initiative dataset.

**Results:**

The multivariate analysis identified significant Time × Treatment Group interactions for knee flexion–extension, pelvic tilt, and back flexion–extension after FDR correction. Gait speed differed between groups (TKR slower, Cohen’s d=−1.02 ) and met Rothman criteria; after adjustment, pelvic tilt retained the lowest uncorrected p -value with no significant Time × Velocity interaction. Random Forest reached a mean ROC-AUC of 0.81 ± 0.06, with pelvic tilt selected in 80% of folds. Six descriptors showed significant associations with joint pain (highest AUC: pelvic tilt 0.80, shoulder rotation 0.81); none reached comparable association with the remaining outcomes. Pelvic tilt showed inverse association with depression and positive association with pain. Adding velocity as a candidate feature did not change Random Forest performance, indicating redundancy with kinematic descriptors at the individual level.

**Conclusion:**

Stance-phase kinematics differentiate two groups of women with moderate KOA who share radiographic severity but follow different treatment pathways. Pelvic tilt emerged as the most consistent descriptor across multivariate, individual-level, and pain-related analyses, and the most robust under adjustment for gait speed. Kinematics offer an accessible alternative to kinetic measurements for describing clinical heterogeneity in moderate KOA, although integration with imaging and patient-reported outcomes is necessary before clinical implementation.

## 1 Introduction

Knee osteoarthritis (KOA) is a musculoskeletal disorder affecting millions of people worldwide, significantly contributing to chronic pain, functional impairment, and diminished quality of life, particularly in aging populations (Konrath et al., 2019; International, 2016; McAlindon et al., 2014). Multiple factors such as age, female sex, and high body mass index (BMI) influence idiopathic KOA (Blagojevic et al., 2010; Silverwood et al., 2015; Shane Anderson and Loeser, 2010; Aspden, 2011). However, its clinical heterogeneity complicates the prediction of disease progression and the optimization of treatment (Bierma-Zeinstra and Verhagen, 2011). Patients sharing the same Kellgren–Lawrence (KL) grade can present very different functional trajectories. Among available interventions, total knee replacement (TKR) is one of the most common orthopaedic procedures worldwide, yet TKR rates vary substantially across regions and health systems, reflecting the extent to which the decision to operate depends on geographic context, clinical culture, and institutional factors beyond disease severity alone (Ward, 2022; Gómez-Barrena et al., 2014). Current decision-making relies primarily on patient-reported outcomes such as the Western Ontario and McMaster Universities Osteoarthritis Index (WOMAC) and on radiographic grading (Faschingbauer et al., 2017; Kahn et al., 2013). Radiography captures structural changes but not the functional or biochemical alterations that may drive symptom severity, and this limited stratification contributes to the 15%–34% of TKR patients who report suboptimal outcomes (Quintana et al., 2006; Bernal-Delgado et al., 2014). Within our own cohort, recent work has shown that patients assigned to TKR presented significantly worse WOMAC, Hospital Anxiety and Depression Scale (HADS), and Pain Catastrophizing Scale scores than those managed conservatively, despite sharing comparable radiographic severity (Ojeda et al., 2025). These findings confirm that clinical and symptomatic measures capture disease heterogeneity that radiography alone does not, yet they also leave open the question of whether objective functional descriptors can provide additional stratification value beyond self-reported scales.

Biomechanical gait analysis offers objective functional descriptors that may complement clinical scales for stratification in KOA. Much of the existing literature has focused on kinetic measures of joint loading, particularly the external knee adduction moment (KAM), which has been associated with radiographic progression of medial compartment KOA over longitudinal follow-up (Miyazaki et al., 2002; Harkey et al., 2021) and correlates with disease severity and varus malalignment in cross-sectional studies (Foroughi et al., 2009). However, the relationship between KAM and clinical symptoms differs across severity subgroups (Hall et al., 2017), and previous work in our own cohort directly addressed the relationship between gait functionality, treatment decision and kinetics in distinct subgroups within the same radiographic severity level. Tassani et al. (2022) analysed ground reaction forces and joint moments in patients matched for KL grade, restricting the analysis to the stance phase as the portion of the gait cycle most relevant to joint loading. Ground reaction forces were not associated with treatment pathway, whereas joint moments showed significant interactions between treatment, age and BMI, and spatiotemporal variables (gait velocity and double-support time) also distinguished the two groups. Because joint moments depend on segment geometry and motion as well as on the applied forces, the finding that the group difference appeared in moments but not in forces suggested that it might reside in the movement patterns themselves, motivating us to examine whether stance-phase kinematics could capture the same differences. We retained the stance phase as the analytical window for consistency with this prior kinetic work and because the sub-phase segmentation used here (loading response, mid-stance, terminal stance) is defined from vertical ground reaction force landmarks that exist only during ground contact.

Kinematic descriptors represent a complementary axis of biomechanical characterization that has gained increasing attention in KOA research (Kaya et al., 2025; Konrath et al., 2019). Compared with controls, individuals with KOA typically walk more slowly with shorter steps, exhibit reduced knee flexion range of motion but increased adduction, and show altered trunk and pelvic coordination (Ro et al., 2019; Kubo et al., 2022; Astephen et al., 2008; Meireles et al., 2016; Halim et al., 2021). Astephen et al. (2008) showed that kinematic and kinetic patterns jointly characterise functional differences across radiographically defined KOA severity groups. Moreover, recent work has shown that kinematic variance is mechanically coupled to the loading patterns that kinetic analyses target. Di Raimondo et al. (2025) applied principal component analysis to gait kinematics in healthy and KOA populations and showed that the dominant modes of kinematic variation (hip flexion–extension and pelvic tilt) drive a substantial part of the variance in model-estimated knee contact forces. These findings suggest that kinematic descriptors capture mechanically relevant information about joint loading, and may therefore serve as informative descriptors for subgroup differentiation. A further practical consideration is that kinematic data can now be acquired with increasingly accessible technologies, including wearable inertial sensors and markerless motion capture systems, which broadens the settings in which such analyses can be applied (Uhlrich et al., 2023). Nevertheless, there is no consensus on which kinematic descriptors are most useful for differentiating clinically meaningful subgroups within the same radiographic severity level, and few studies have addressed this question in women specifically. A further consideration is that gait speed modulates kinematic patterns and should be accounted for in group comparisons to avoid confounding (Mills et al., 2013; Landry et al., 2007).

The aim of this study was to evaluate whether stance-phase kinematic descriptors carry clinical relevance for stratifying women with moderate KOA (KL grades 2–3), assessed through their association with patient-reported outcomes involved in treatment decision-making and through their ability to differentiate between two subgroups—those scheduled for TKR and those managed conservatively. We hypothesized that specific kinematic trajectories across stance sub-phases would differ between these subgroups and that the identified descriptors would associate with patient-reported outcomes. Given that gait speed modulates kinematic patterns (Astephen et al., 2008), we additionally examined its relationship with between-group differences to determine whether it acts as a confounder or as a modifier of kinematic variation. A secondary aim was to identify the most influential descriptors through a machine-learning approach, to assess whether the two methods highlight the congruent or complementary aspects of gait.

## 2 Methods

### 2.1 Study design and participants

This cross-sectional study analyzed data from the HOLOA/STRATO cohort^1^, an ongoing prospective recruitment conducted at Hospital del Mar (Barcelona, Spain) since 2016. Women aged 60–75 years with a clinical and radiographic diagnosis of moderate KOA (Kellgren–Lawrence grades 2–3) were enrolled by a physician and a nurse experienced in osteoarthritis assessment. Balanced recruitment of male participants was challenging because prior meniscectomy was an exclusion criterion and was particularly prevalent among patients belonging to the TKR group. Thus, only females were retained to avoid sex confounding (Tassani et al., 2022) and to ensure a well-matched case-control cohort. Participants with evidence of secondary KOA were excluded an full eligibility criteria are detailed in Table 1. The study followed the Declaration of Helsinki and Good Clinical Practice guidelines. The protocol received approval from the Clinical Research Ethical Committee at Hospital del Mar (references 2016/6747/I and 2022/10509/I). All participants signed informed consent and agreed with specified washout periods for their treatments: 3 months for intra-articular hyaluronic acid, 1 month for corticosteroids, 48 h for oral anti-inflammatory drugs, and 24 h for analgesics.

**TABLE 1:** Inclusion and exclusion criteria for participant recruitment.

Inclusion criteria
Adults between 60 and 75 years able to understand study information and give informed consent
Diagnosis of KOA in at least one knee (American college of rheumatology criteria) (Bellamy et al., 1999)
Stable KOA symptoms for ≥ 3 months before recruitment
Knee radiography (performed in the previous 12 months) graded 2 or 3 according to the Kellgren–Lawrence (KL) scale

Exclusion criteria
Secondary KOA (trauma, meniscectomy, overuse, inflammatory or connective tissue disease)
Pathological misalignment (more than 5°)
Need for walking aids (cane, walker, or crutches)
Unstable medical conditions that may interfere with gait or other study outcomes
History or current substance abuse within the previous 6 months
Pregnancy or lactation
Contra-indications to magnetic resonance imaging (metallic implants, claustrophobia, contrast allergy)
Ongoing treatment
Oral anti-inflammatory drugs within 48 h before gait testing
Analgesics within 24 h before gait testing
Intra-articular corticosteroid injection within 3 months
Intra-articular hyaluronic acid injection within 6 months

Participants were classified into two clinical subgroups according to the treatment strategy already indicated by their treating physician prior to study enrollment: conservative management (pharmacological treatment, physical therapy, or lifestyle modifications) or total knee replacement (TKR). Patients included in the TKR subgroup had already been placed on the surgical waiting list at the time of recruitment. Although both groups share the same radiographic severity range (KL 2–3), they differ in clinical and functional profiles previously characterized in HOLOA cohort (Ojeda et al., 2025). The treatment label is used in this study as a descriptor of clinical and functional heterogeneity within moderate KOA, rather than as a prediction target for the surgical decision itself, since the decision is shared between patient and clinician (see protocol at Supplementary Material).

To reduce the confounding effects of known KOA risk factors, the HOLOA cohort applied stratified recruitment by age (60–67 vs. 68–75 years) and BMI (non-obese <30 kg/m^2^ vs. obese ≥30 kg/m^2^); the STRATO cohort recruited participants stratified by treatment pathway only. Although no formal matching on KL grade was performed, *post hoc* comparisons confirmed no significant between-group differences in KL grade across any of the three compartments or in the global score, minimizing confounding by indication (Kyriacou and Lewis, 2016).

### 2.2 Gait data acquisition and preprocessing

Gait analysis was performed with an eight-camera motion capture system (1.5 Mpixel, 250 fps; BTS SmartDX 700, BTS Engineering, Milan, Italy) and two synchronized force plates (500 Hz; BTS P-6000). The Helen Hayes marker protocol (Davis et al., 1991), including medial markers for static calibration, was used for 3D gait assessment. Twenty-two reflective markers were placed on the trunk, pelvis, thighs, shanks, and feet, following the protocol described in Tassani et al. (2022). After static calibration, medial markers were removed and each participant performed at least five valid barefoot trials along a 10-m walkway at self-selected speed. Five trials per subject were averaged to generate a representative gait cycle. Self-selected (habitual) walking speed was recorded for each participant and used in subsequent analyses as a descriptor of functional capacity.

**FIGURE 1:**
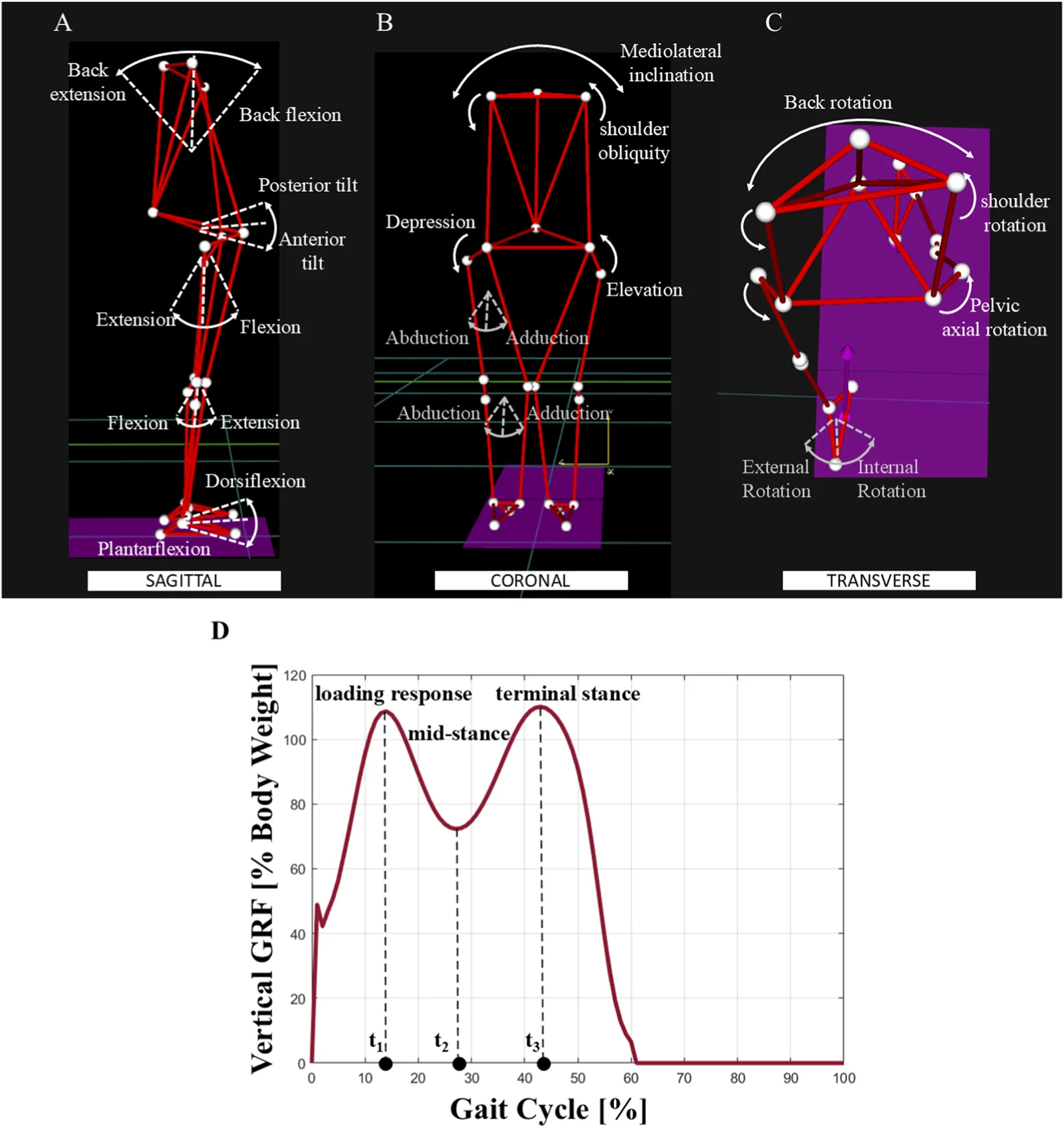
Articular rotations and definition of the stance sub-phases within the gait cycle. Articular rotations shown in **(A)** the sagittal plane, **(B)** the coronal plane, and **(C)** the transverse plane. Solid arcs and white labels indicate the twelve kinematic variables retained for analysis; dashed grey arcs indicate the five variables excluded because of the measurement limitations of the Helen Hayes marker set for frontal- and transverse-plane knee, hip, and ankle rotations. Shoulder tilt (sagittal-plane rotation of the shoulder line) is defined in Methods 2.3 and is not depicted graphically due to the lateral view of **(A)**. **(D)** Vertical ground reaction force during a representative gait cycle, normalized to body weight. The three dashed vertical lines identify the analysis points corresponding to loading response $\left(t_{1}\right)$ , mid-stance $\left(t_{2}\right)$ , and terminal stance $\left(t_{3}\right)$ , defined as the time points at which the first derivative of the vertical ground reaction force equals zero. Marker placement followed the protocol described in Tassani et al. (2022).

Ground reaction forces and kinematic data were processed in Smart Analyzer software (BTS Engineering). Joint angles (Figures 1a–c) analyzed included flexion–extension (FE), abduction–adduction (AA), and internal–external rotation (IE) of the knee and hip; ankle FE; pelvic obliquity, tilt, and axial rotation; back mediolateral inclination, FE, and IE; and shoulder obliquity, tilt, and rotation (offset-corrected). Gait cycles were normalized from 0% (heel strike) to 100% (subsequent heel strike). The stance phase (Figure 1d) was segmented into loading response $\left(t_{1}\right)$ , mid-stance $\left(t_{2}\right)$ , and terminal stance $\left(t_{3}\right)$ based on the time points when the first derivative of the vertical ground reaction force equaled zero (Tassani et al., 2022).

These three sub-phases ( $t_{1}$ , $t_{2}$ , $t_{3}$ ) constitute the within-subject factor referred to as *Time* in all subsequent repeated-measures analyses, capturing the progression of each kinematic variable across discrete stance sub-phases rather than elapsed time.

### 2.3 Selection of kinematic variables

Of the 17 joint angles recorded, five were excluded *a priori* from all statistical analyses because of documented measurement limitations of the Helen Hayes marker set: knee internal–external rotation (KIE), knee abduction–adduction (KAA), hip abduction–adduction (HPAA), hip internal–external rotation (HPIE), and ankle internal–external rotation (AIE). Soft tissue artifact affects transverse-plane rotations and frontal-plane knee angles to a substantially greater extent than sagittal-plane variables, with reported errors of 4°–8° versus 1°–2°, respectively (Cappozzo et al., 2005; Benoit et al., 2006; Wang et al., 2023). In particular, KAA is sensitive to thigh marker alignment and shows sagittal-plane crosstalk that can exceed the actual movement range. This decision was made before examining any group-level result and is therefore not a *post hoc* sensitivity analysis. The twelve retained variables were: ankle FE (AFE), knee FE (KFE), hip FE (HPFE), pelvic tilt (PTILT), pelvic obliquity (POBLI), pelvic axial rotation (PROT), back mediolateral inclination (SPML), back internal–external rotation (SPIE), back FE (SPFE), and shoulder tilt, obliquity, and rotation offset (SHTILTOFF, SHOBLIOFF, SHROTOFF). Results with the full 17-variable set are reported for completeness in Supplementary Material.

The retained set deliberately included markers from the trunk-pelvis segment (back flexion-extension, mediolateral inclination, internal-external rotation; pelvic tilt, obliquity, and axial rotation) and the upper body (shoulder tilt, obliquity, and axial rotation), in addition to the lower-limb sagittal angles. Trunk and pelvic kinematics during gait in KOA have been documented as compensatory adaptations to limited knee extension, with reports of greater anterior pelvic tilt, forward trunk flexion, and lateral trunk lean in patients with more severe disease (Suzuki et al., 2023; Preece et al., 2019). Trunk-pelvis position also modulates the external moments transmitted to the knee through its effect on the location of the body’s center of mass (Suzuki et al., 2023). Shoulder kinematics during walking, which include arm swing and axial rotation, contribute to dynamic balance and postural control, and have been characterized as sensitive to age-related and pathological gait changes in older populations (Mirelman et al., 2015; Hill and Nantel, 2019). Including the upper-body and trunk-pelvis segments therefore allowed the analysis to capture compensatory mechanisms that operate above the affected joint and that may differentiate patients with similar radiographic severity but distinct functional profiles.

### 2.4 Statistical analysis

A correlational screening of the 12 retained variables across the three stance sub-phases (36 variables in total) was first conducted to reduce dimensionality by identifying redundant features (Pearson’s |r|>0.9 ). Analyses were performed in SPSS version 28 (IBM Corp., NY, United States).

#### 2.4.1 Gait speed characterization and confounding assessment

Before the multivariate analysis, self-selected gait speed was evaluated as a potential confounder of the group-level kinematic comparison following the criteria described by Rothman et al. (2008). Criterion 1 (association with group allocation) was tested by comparing group means with Welch’s t-test, after assessing normality with Shapiro–Wilk and variance homogeneity with Levene’s test. Effect size was reported as Cohen’s d with 95% bootstrap confidence intervals. Criterion 2 (association with the kinematic outcomes) was assessed with Pearson correlations between gait speed and each of the 12 kinematic variables at $t_{1}$ , $t_{2}$ , $t_{3}$ , and the value of the change between $t_{3}$ and $t_{1}$ ( $D={\text{Angle}}_{t_{3}}-{\text{Angle}}_{t_{1}}$ ; full correlation results are reported in Supplementary Material. Velocity was considered a formal confounder when both criteria were met.

#### 2.4.2 Model A—repeated-measures analysis without covariate

A repeated-measures multifactorial MANOVA was first conducted with the three stance sub-phases ( $t_{1}$ , $t_{2}$ , $t_{3}$ ) as within-subject factor and treatment subgroup, age category, and BMI category as between-subject factors. Multivariate effects were reported with Pillai’s Trace, F-statistic, p -value, and partial eta-squared $\left(\eta_{p}^{2}\right)$ . Significant multivariate effects were followed by univariate ANOVAs to identify the variables driving the interaction. Sphericity was assessed with Mauchly’s test, and Greenhouse–Geisser corrections were applied when the assumption was violated. To account for multiple comparisons across the 12 univariate Time × Subgroup tests, false discovery rate (FDR) correction was applied using the Benjamini–Hochberg procedure. This model describes the Time × Treatment Group effect and serves as the reference for evaluating the impact of confounder adjustment in Model B.

#### 2.4.3 Model B—repeated-measures analysis adjusted for gait speed

Model A was extended by adding self-selected gait speed as a continuous between-subject covariate, producing a repeated-measures MANCOVA. Because gait speed met both Rothman criteria for confounding (see Section 3.2), this model provides the confounder-adjusted estimate of the Time × Treatment Group effect. The Time × Velocity interaction was additionally examined to characterize whether walking speed modulates kinematic trajectories across the stance phase. The contrast between Models A and B was interpreted as the extent to which the unadjusted Time × Treatment Group effect is attributable to between-group differences in gait speed. FDR correction was applied within each model across the 12 univariate tests. We report both uncorrected and FDR-corrected p -values for transparency. The statistical threshold was α=0.05 throughout.

### 2.5 Random forest classification with exhaustive feature selection

As a complementary analysis, a Random Forest (RF) classifier was trained to evaluate whether stance-phase kinematic changes carry subgroup-discriminative signals at the individual level. For each of the 12 variables, the value of the angle between $t_{3}$ and $t_{1}$ $D={\text{Angle}}_{t_{3}}-{\text{Angle}}_{t_{1}}$ was computed. The RF was trained to distinguish the two clinical subgroups (conservative vs. TKR) using nested 5-fold cross-validation. To minimise overfitting and optimism bias, all hyperparameter tuning (via randomized search; full details in Supplementary Material) and feature selection (via exhaustive feature selection, EFS) were performed within the inner loop using stratified folds, ensuring that the outer test folds remained completely independent from model development. This nested design follows best-practice recommendations for unbiased performance estimation when feature selection and hyperparameter tuning are combined, particularly in small-sample settings (Vabalas et al., 2019; Hastie, 2009). EFS tested all possible feature subsets up to a maximum of five features. This limit was introduced to constrain model complexity given the available sample size and was guided by the events-per-variable (EPV) principle. With the smaller class (n = 27) as the limiting factor, five features yield an EPV = 5.4, which sits within the 5–9 EPV range considered acceptable in exploratory predictive analyses (Vittinghoff and McCulloch, 2007), while remaining consistent with the small-sample constraints described by Peduzzi et al. (1996). Fold-specific ROC curves were generated from predicted probabilities, and the mean ROC-AUC across outer folds was reported as the primary performance metric, with accuracy, precision, recall, specificity, F1-score, balanced accuracy, and Matthews correlation coefficient reported as complementary metrics (mean ± SD across folds). AUC values close to 0.5 indicate absence of discrimination, whereas values clearly below 0.5 indicate an inverse association between the kinematic descriptor and the clinical outcome. Feature stability was assessed as the proportion of outer folds in which each variable was retained by EFS. This analysis is intended to test whether the same kinematic dimensions that emerge from the repeated-measures analysis also support individual-level discrimination, and whether the two methods highlight the same or complementary aspects of gait.

### 2.6 Associations between kinematic descriptors and clinical variables

To explore the clinical relevance of the stance-phase kinematic descriptors, the association between each of the 12 reliable D variables and a set of binarized clinical outcomes (joint pain, rigidity, functional limitation, depression, central sensitization, catastrophism, effusion, and synovitis) was assessed using univariate logistic regression. Clinical thresholds for binarization were those calibrated and validated for the HOLOA cohort in Segarra-Queralt et al. (2024). Each model was evaluated with repeated stratified 10-fold cross-validation (three repetitions); cross-validated ROC-AUC was used as the descriptive performance metric. Statistical significance of each association was assessed via the p -value of the regression coefficient, and the FDR was controlled within each clinical outcome (Benjamini–Hochberg correction over 12 kinematic variables per outcome).

### 2.7 Clinical construct consistency against the OAI cohort

To assess the generalizability of the clinical constructs underlying treatment decisions in our cohort, WOMAC pain, rigidity, and functionality subscales were compared between HOLOA/STRATO and the Osteoarthritis Initiative (OAI) dataset. OAI participants were filtered for female sex, age 60–75 years, BMI 17–40 kg/m^2^, KL grades 2–3, no prior knee surgeries or injuries, and complete WOMAC data; for bilateral cases, the primary leg was selected. Normality and homogeneity of variances were assessed, and between-cohort differences tested with Mann–Whitney U test due to violations of normality. Within-cohort subscale associations were examined with Pearson correlations, and between-cohort correlation differences tested with Fisher r-to-z transformations. A classification model based only on WOMAC subscales was additionally trained to benchmark the discriminative capacity of patient-reported outcome measures (PROM) alone. This analysis does not constitute external validation of the kinematic findings, since the OAI dataset does not contain kinematic angle measurements; its purpose is to document whether the clinical constructs that underpin treatment decisions behave consistently across populations, and thus whether the limitations of PROM-only stratification generalize beyond our cohort. Full results are reported in Supplementary Material.

## 3 Results

### 3.1 Participant characteristics

Of 128 participants screened, 66 women met the inclusion criteria and were retained for analysis: 27 in the TKR group and 39 in the conservative group. Mean age was 67.4 years ± 5.2 in the conservative group and 66.7 years ± 4.0 in the TKR group. Mean BMI was 30.1 kg/m^2^ ± 4.9 and 31.4 kg/m^2^ ± 5.1, respectively. Although both groups share the same radiographic severity range (KL 2–3), they differ in clinical and functional profiles, as previously documented in the HOLOA sub-cohort, where the TKR group reported higher WOMAC pain, stiffness, and functional limitation scores, and higher HADS scores than the conservative group (Ojeda et al., 2025).

### 3.2 Gait speed comparison and confounding assessment

Self-selected gait speed differed significantly between groups (TKR: 0.77±0.18 m/s; conservative: 0.96±0.18 m/s; Welch t(56.8)=−4.083 , p<.001 ; Cohen’s d=−1.02 , 95% bootstrap CI ( −1.69 , −0.49 )), with a large effect size and the TKR group walking slower than the conservative group. Shapiro–Wilk and Levene’s tests supported the use of a parametric comparison (all p>0.29 ). Rothman’s Criterion 1 (association with group allocation) was therefore met. Pearson correlations between gait speed and the 12 kinematic variables at $t_{1}$ , $t_{2}$ , $t_{3}$ , and $D={\text{Angle}}_{t_{3}}-{\text{Angle}}_{t_{1}}$ are reported in Supplementary Material; 12 of 48 correlations had |r|>0.3 with p<.05 , the strongest being DKFE (r=−0.59) and DPOBLI (r=−0.59) . Rothman’s Criterion 2 was met for these 12 variables. Because both criteria were satisfied, gait speed was treated as a formal confounder of the Time × Treatment Group effect and included as a continuous covariate in Model B.

### 3.3 Model A—repeated-measures MANOVA without velocity

The multivariate repeated-measures analysis showed a significant Time × Treatment Group interaction (Pillai’s Trace = 0.495, F = 2.907, p -value < 0.001, Partial $\eta^{2}$ = 0.248, Power = 1.000), indicating that stance-phase kinematic trajectories differed between groups (Table 2). For univariate repeated-measures ANOVAs, sphericity was violated in all cases (Mauchly’s test, all p<.007 ; Supplementary Material) and Greenhouse–Geisser corrections were applied where appropriate. Univariate tests with FDR correction identified three variables driving this interaction (Table 3): knee flexion/extension (KFE), pelvic tilt (PTILT), and back flexion/extension (SPFE). Ankle FE was significant uncorrected ( p = 0.037) but did not survive FDR ( $p_{\mathrm{FDR}}$ = 0.111). The remaining eight variables showed no significant Time × Group interaction.

**TABLE 2:** Multivariate tests for within-subjects effects from the repeated-measures multifactorial MANOVA (MODEL A).

Effect	Pillai’s trace	*F*	*p*-value	Partial $\eta^{2}$	Observed power
Time point	**1.743**	**59.870**	< **0.001**	**0.871**	**1.000**
Time point × treatment group	**0.495**	**2.907**	< **0.001**	**0.248**	**1.000**
Time point × age group	0.290	1.497	0.070	0.145	0.952
Time point × BMI group	**0.340**	**1.807**	**0.015**	**0.170**	**0.984**
Time point × treatment group × age group	0.233	1.163	0.279	0.116	0.865
Time point × treatment group × BMI group	0.195	0.956	0.526	0.098	0.766
Time point × age group × BMI group	0.275	1.406	0.106	0.137	0.936
Time point × treatment group × age group × BMI group	**0.366**	**1.980**	**0.006**	**0.183**	**0.992**

The table displays the multivariate test statistics (Pillai’s Trace) assessing changes across stance-phase time points and their interactions with treatment, age, and BMI groups. The *F* statistic and corresponding *p*-value denote the significance of each effect; *Partial* $\eta^{2}$ represents the multivariate effect size, and *Observed Power* indicates the probability of detecting a true effect given the sample size and observed variance. Statistically significant results (*p* < 0.05) are shown in bold.

**TABLE 3:** Univariate within-subjects results for the Time × Treatment Group interaction (Model A).

Variable	*F*	*p*-value (GG)	*p* (FDR)	Partial $\eta^{2}$
KFE	**9.444**	**0.001**	**0.006**	**0.140**
PTILT	**10.323**	**0.001**	**0.006**	**0.151**
SPFE	**7.966**	**0.004**	**0.016**	**0.121**
AFE	4.062	0.037	0.111	0.065
POBLI	2.968	0.068	0.163	0.049
SHTILTOFF	2.765	0.090	0.180	0.046
SHROTOFF	2.354	0.127	0.218	0.039
SPIE	1.290	0.268	0.402	0.022
HPFE	1.028	0.322	0.429	0.017
SHOBLIOFF	0.488	0.555	0.666	0.008
PROT	0.176	0.736	0.784	0.003
SPML	0.122	0.784	0.784	0.002

AFE: ankle flexion-extension; KFE: knee flexion-extension; HPFE: hip flexion-extension; PTILT: pelvic anterior-posterior tilt; POBLI: pelvic obliquity; PROT: pelvic axial rotation; SPML: back mediolateral inclination; SPIE: back internal-external rotation; SHTILTOFF: shoulder tilt; SHOBLIOFF: shoulder obliquity; SHROTOFF: shoulder axial rotation; SPFE: back flexion-extension.

*F*-values and *p*-values were obtained with the Greenhouse-Geisser correction for sphericity violations. *Partial* $\eta^{2}$ represents the univariate effect size. *p*-values are reported uncorrected and after Benjamini-Hochberg false discovery rate (FDR) correction applied across the twelve variables. Statistically significant results after FDR correction (*p* < 0.05) are shown in bold.

The multivariate analysis also identified a significant Time × BMI interaction (Pillai’s trace = 0.340, F = 1.807, p = 0.015, $\eta_{p}^{2}$ = 0.170) and a four-way Time × Treatment Group × Age × BMI interaction (Pillai’s trace = 0.366, F = 1.980, p = 0.006, $\eta_{p}^{2}$ = 0.183), indicating that stance-phase kinematic trajectories vary with anthropometric and demographic characteristics. However, at the univariate level, no individual variable remained significant after FDR correction for either interaction; full results are presented in Supplementary Material.

### 3.4 Model B—repeated-measures MANCOVA adjusted for gait speed

When gait speed was included as a covariate, the multivariate Time × Treatment Group interaction was attenuated and no longer reached significance (Pillai’s trace = 0.262, F = 1.309, p = 0.125, $\eta_{p}^{2}$ = 0.131), indicating that a substantial portion of the unadjusted Model A effect is attributable to between-group differences in gait speed. A large Time × Velocity interaction emerged (Pillai’s trace = 0.735, F = 5.040, p<0.001 , $\eta_{p}^{2}$ = 0.368), see Table 4. The between-subjects effect of velocity was not significant (Pillai’s trace = 0.166, F = 0.764, p = 0.682, $\eta_{p}^{2}$ = 0.166), and no individual variable showed a significant univariate between-subjects velocity effect after FDR correction.

**TABLE 4:** Multivariate tests for within-subjects effects from the repeated-measures MANCOVA (Model B), including gait speed (*mean velocity*) as a covariate. Pillai’s Trace is reported for each effect.

Effect	Pillai’s trace	*F*	*p*-value	Partial $\eta^{2}$	Observed power
Time point	**0.372**	**1.982**	**0.006**	**0.186**	**0.992**
Time point × *mean velocity*	**0.735**	**5.040**	< **0.001**	**0.368**	**1.000**
Time point × treatment group	0.262	1.309	0.160	0.131	0.912
Time point × age group	0.212	1.030	0.429	0.106	0.806
Time point × BMI group	0.227	1.108	0.337	0.113	0.842
Time point × treatment group × age group	0.240	1.180	0.263	0.120	0.871
Time point × treatment group × BMI group	0.195	0.937	0.552	0.098	0.754
Time point × age group × BMI group	0.274	1.373	0.123	0.137	0.928
Time point × treatment group × age group × BMI group	**0.380**	**2.032**	**0.004**	**0.190**	**0.993**

All tests reported with hypothesis df = 24 and error df = 208.

The *F* statistic and corresponding *p*-value denote the significance of each effect; *Partial* $\eta^{2}$ represents the multivariate effect size, and *Observed Power* indicates the probability of detecting a true effect given the sample size and observed variance. Statistically significant results (*p* < 0.05) are shown in bold.

Univariate Time × Treatment Group tests showed that no variable survived FDR correction in Model B. PTILT was the only variable significant at the uncorrected level ( p = 0.010, $\eta_{p}^{2}$ = 0.099; $p_{\mathrm{FDR}}$ = 0.120), see Table 5. Four variables showed Time × Velocity interactions surviving FDR (KFE, POBLI, AFE, SPFE), while PTILT did not show a significant Time × Velocity interaction ( p = 0.694), see Table 6.

**TABLE 5:** Univariate within-subjects results for the Time × Treatment Group interaction (Model B, with *mean velocity* as covariate) for the twelve kinematic variables retained after excluding variables with high measurement error in the Helen Hayes marker set (AIE, KIE, KAA, HPAA, HPIE).

Variable	*F*	*p*-value (GG)	*p* (FDR)	Partial $\eta^{2}$
PTILT	**6.283**	**0.010**	0.120	**0.099**
SHTILTOFF	2.093	0.147	0.599	0.035
SPFE	1.509	0.227	0.599	0.026
POBLI	1.229	0.293	0.599	0.021
KFE	1.199	0.294	0.599	0.021
SPML	1.128	0.307	0.599	0.019
AFE	0.823	0.397	0.599	0.014
SPIE	0.654	0.448	0.599	0.011
SHROTOFF	0.610	0.453	0.599	0.011
PROT	0.555	0.499	0.599	0.010
SHOBLIOFF	0.206	0.744	0.812	0.004
HPFE	0.037	0.872	0.872	0.001

AFE: ankle flexion-extension; KFE: knee flexion-extension; HPFE: hip flexion-extension; PTILT: pelvic anterior-posterior tilt; POBLI: pelvic obliquity; PROT: pelvic axial rotation; SPML: back mediolateral inclination; SPIE: back internal-external rotation; SHTILTOFF: shoulder tilt; SHOBLIOFF: shoulder obliquity; SHROTOFF: shoulder axial rotation; SPFE: back flexion-extension.

*F*-values and *p*-values were obtained with the Greenhouse-Geisser correction for sphericity violations. *Partial* $\eta^{2}$ represents the univariate effect size. FDR correction was applied across the twelve variables using the Benjamini-Hochberg procedure. No variable survived FDR correction. Statistically significant uncorrected results (*p* < 0.05) are shown in bold.

**TABLE 6:** Univariate within-subjects results for the Time × *mean velocity* interaction (Model B) for the twelve kinematic variables retained after excluding variables with high measurement error in the Helen Hayes marker set (AIE, KIE, KAA, HPAA, HPIE).

Variable	*F*	*p*-value (GG)	*p* (FDR)	Partial $\eta^{2}$
KFE	**14.146**	< **0.001**	< **0.001**	**0.199**
POBLI	**14.058**	< **0.001**	< **0.001**	**0.198**
AFE	**7.104**	**0.005**	**0.018**	**0.111**
SPFE	**7.525**	**0.006**	**0.018**	**0.117**
HPFE	4.046	0.044	0.098	0.066
SPML	3.703	0.049	0.098	0.061
SHOBLIOFF	2.167	0.134	0.230	0.037
SHROTOFF	2.092	0.151	0.226	0.035
PROT	0.595	0.481	0.641	0.010
PTILT	0.396	0.578	0.694	0.007
SPIE	0.174	0.723	0.789	0.003
SHTILTOFF	0.117	0.801	0.801	0.002

AFE: ankle flexion-extension; KFE: knee flexion-extension; HPFE: hip flexion-extension; PTILT: pelvic anterior-posterior tilt; POBLI: pelvic obliquity; PROT: pelvic axial rotation; SPML: back mediolateral inclination; SPIE: back internal-external rotation; SHTILTOFF: shoulder tilt; SHOBLIOFF: shoulder obliquity; SHROTOFF: shoulder axial rotation; SPFE: back flexion-extension.

*F*-values and *p*-values were obtained with the Greenhouse-Geisser correction for sphericity violations. *Partial* $\eta^{2}$ represents the univariate effect size. FDR correction was applied across the twelve variables using the Benjamini-Hochberg procedure. Statistically significant results after FDR correction (*p* < 0.05) are shown in bold.

### 3.5 Random Forest classification

**FIGURE 2:**
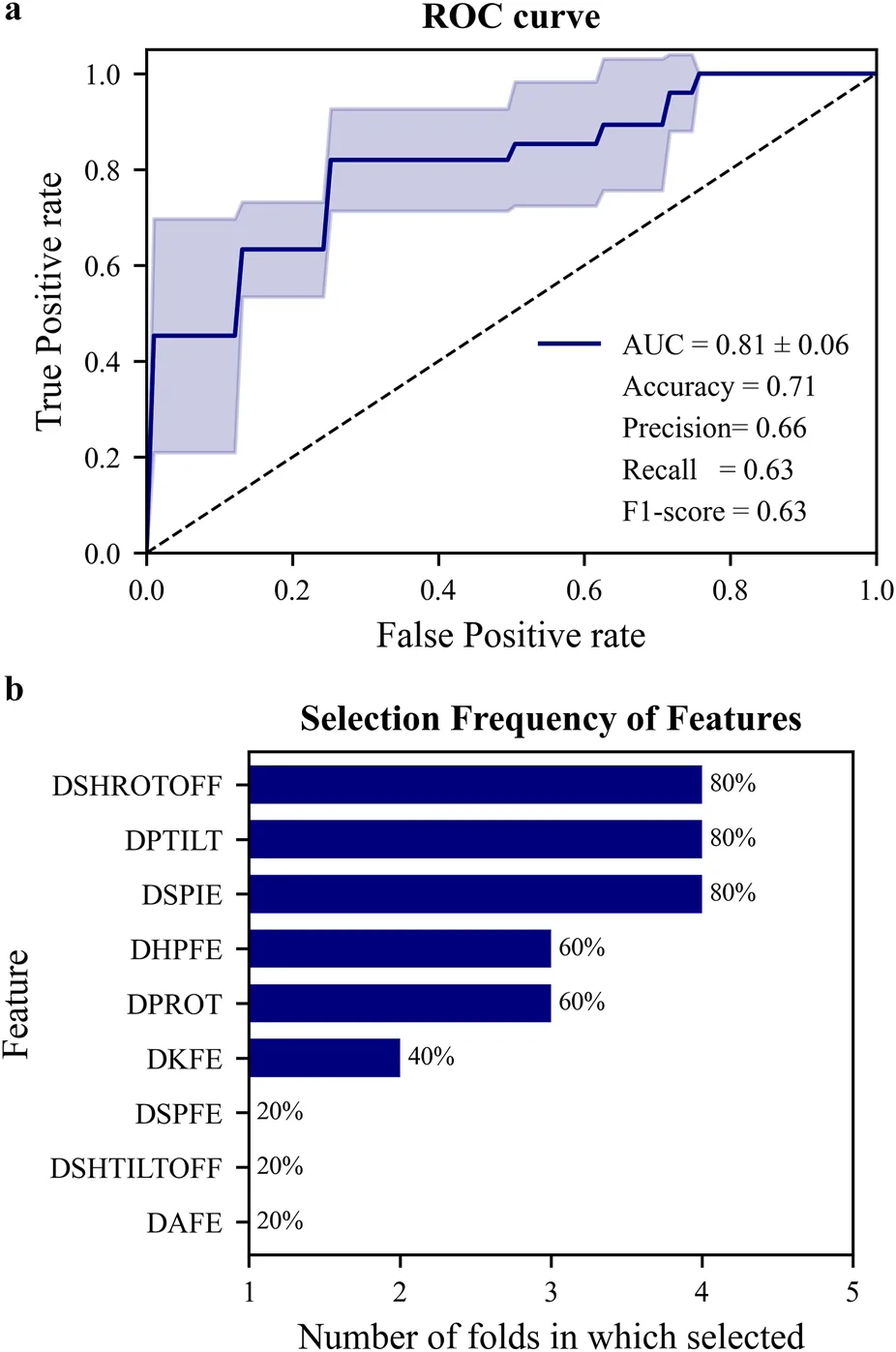
Results from nested cross-validation with exhaustive feature selection (EFS) using a Random Forest classifier. **(a)** Mean Receiver Operating Characteristic (ROC) curve obtained through nested cross-validation ( 5×5 folds). The blue line represents the mean ROC curve across the five outer folds, while the shaded area indicates ± 1 standard deviation of the true positive rate. The dashed diagonal line corresponds to random classification. The average area under the curve (AUC) is shown with its standard deviation (AUC = 0.81 ± 0.06). **(b)** Frequency of feature selection across outer folds during the nested cross-validation with exhaustive feature selection using a Random Forest classifier. Each bar represents the number of folds (out of five) in which a given feature was selected as part of the optimal subset. Features appearing in multiple folds indicate higher selection stability and potential relevance for model robustness.

The RF classifier achieved a mean ROC-AUC of 0.81±0.06 across the five outer folds, with accuracy 0.71, precision 0.66, recall 0.63, F1-score 0.63, specificity 0.77, balanced accuracy 0.70, and a Matthews correlation coefficient 0.43. Fold-level performance metrics are reported in the Supplementary Material, and the mean ROC curve is shown in Figure 2a.

The most frequent feature subset across folds included DKFE, DPROT, DPTILT, DSHROTOFF, and DSPIE and feature stability across the five outer folds are both found in Figure 2b). A configuration with EFS capped at three features produced lower AUC and higher variance (0.76±0.11) and is reported in Supplementary Material S5.

Model A and the RF identified partially overlapping sets of discriminative variables. DPTILT was among the most stable features in the RF and among the FDR-surviving variables in Model A. DKFE, which showed Time × Treatment Group effect in Model A, was selected by EFS in 40% of folds. DSPFE, also a Model A survivor, was selected in only 20% of folds. DSPIE and DSHROTOFF were stable RF features but did not show significant Time × Group effects in Model A.

As a sensitivity analysis, the Random Forest was re-trained with velocity included as an additional candidate feature alongside the 12 stance-phase change variables. Velocity was not selected in any of the cross-validation folds, and overall classification performance was essentially unchanged (AUC = 0.80 ± 0.08), indicating that velocity does not contribute discriminative information beyond the kinematic descriptors at the individual level (full details in Supplementary Material).

### 3.6 Associations between kinematic descriptors and clinical variables

**FIGURE 3:**
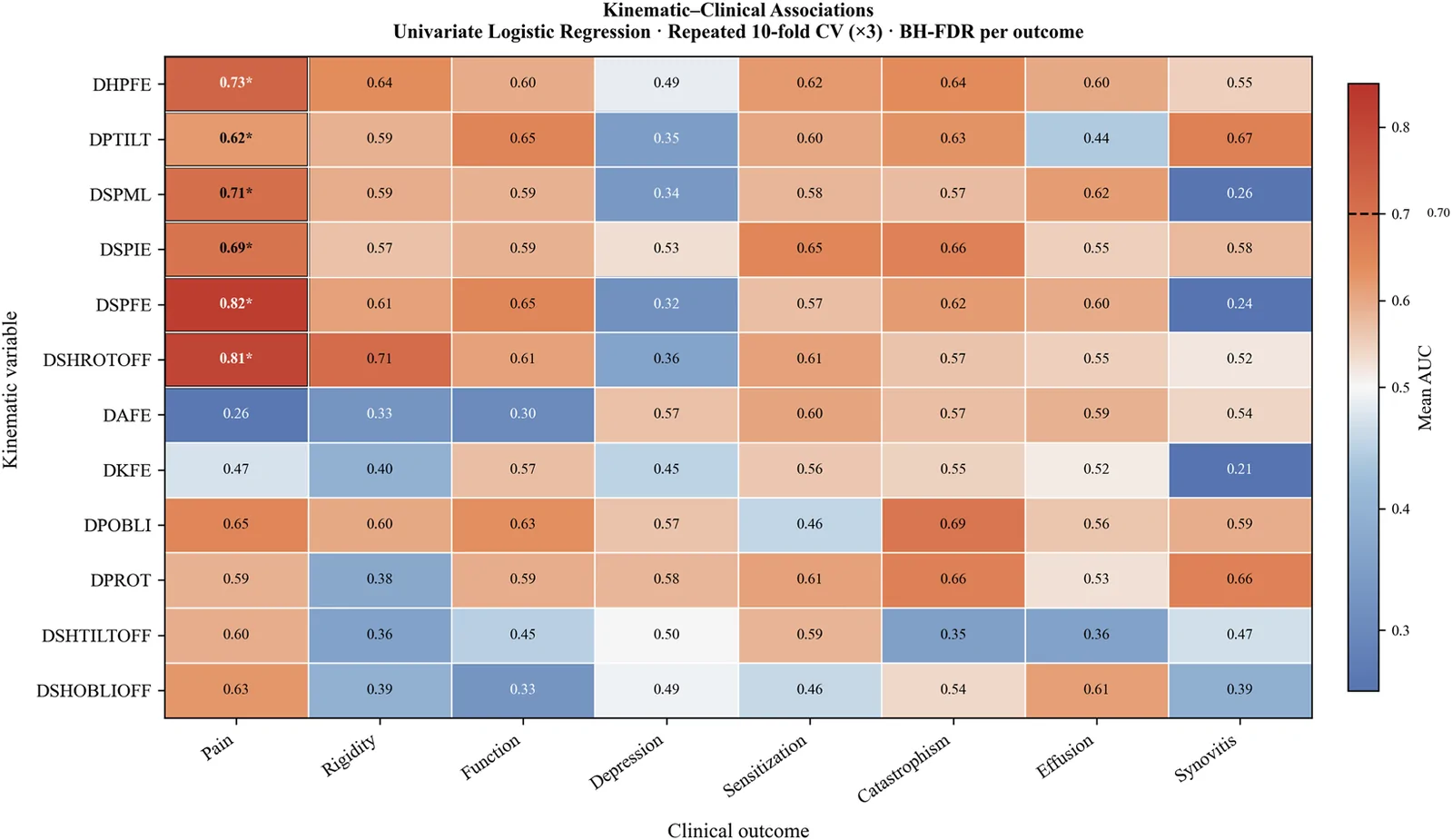
Heatmap of univariate associations between kinematic variables and clinical outcomes. Each cell displays the mean AUC (area under the ROC curve) obtained from univariate logistic regression models evaluated via repeated 10-fold cross-validation (3 repetitions). Rows represent kinematic variables, sorted in descending order by the number of statistically significant associations. Columns represent clinical outcomes. The diverging colormap is centred at AUC = 0.50 (chance level), with warmer colours (orange–red) indicating stronger discriminative performance and cooler colours (blue) indicating performance below chance. Cells outlined in black and marked with an asterisk (*) indicate associations that survived Benjamini–Hochberg false discovery rate (FDR) correction at q<0.05 , applied independently per outcome. The dashed line on the colorbar marks the AUC = 0.70 threshold, used as a reference for clinically relevant discrimination.

The associations between the 12 stance-phase kinematic change variables $\left(D={\text{Angle}}_{t_{3}}-{\text{Angle}}_{t_{1}}\right)$ and the eight binarized clinical outcomes are summarized in Figure 3. Full numerical results, including AUCs with 95% confidence intervals, uncorrected p -values, and FDR-corrected p -values within each clinical outcome, are reported in Supplementary Table S12.

Six associations survived FDR correction, all with joint pain: DSHROTOFF (AUC = 0.81, $p_{\mathrm{FDR}}$ = 0.002), DPTILT (AUC = 0.80, $p_{\mathrm{FDR}}$ = 0.002), DSPIE (AUC = 0.72, $p_{\mathrm{FDR}}$ = 0.006), DHPFE (AUC = 0.71, $p_{\mathrm{FDR}}$ = 0.034), DSPML (AUC = 0.70, $p_{\mathrm{FDR}}$ = 0.034), and DSPFE (AUC = 0.65, $p_{\mathrm{FDR}}$ = 0.011). At the descriptive AUC ≥ 0.70 threshold, two additional associations were observed: DPTILT with functional limitation (AUC = 0.73) and DSPFE with rigidity (AUC = 0.71), both not surviving FDR.

No kinematic descriptor reached AUC ≥ 0.70 for depression, sensitization, catastrophism, effusion, or synovitis. Mean AUC across the 12 kinematic variables was highest for joint pain (0.64) and functional limitation (0.62), and lower for the remaining outcomes (range 0.48–0.56). Several kinematic descriptors showed AUC values consistently below 0.40, indicating an inverse association with the corresponding clinical outcome (i.e., the kinematic descriptor predicted the absence of the outcome). The most pronounced inverse associations were observed for depression: DPTILT, DHPFE, DSHROTOFF, and DSHOBLIOFF all reached AUC < 0.34 for depression while showing AUC > 0.66 for joint pain.

### 3.7 Clinical construct consistency against the OAI cohort

After preprocessing the OAI dataset, a total of 447 patients were retained for analysis. Normality and homoscedasticity tests indicated that the OAI cohort violated parametric assumptions; therefore, Mann–Whitney U tests were used for comparison. There were significant differences across all subscales (all p< 0.001; Supplementary Material), with our HOLOA/STRATO cohort exhibiting higher levels of pain, stiffness, and functional impairment. Correlation analyses within each cohort revealed consistent patterns: pain and functionality were strongly correlated (HOLOA/STRATO 0.835; OAI 0.848), while pain–rigidity (HOLOA/STRATO 0.531; OAI 0.657) and rigidity–functionality (HOLOA/STRATO 0.616; OAI 0.713) showed moderate correlations. Fisher r-to-z comparisons showed there was no differences in correlation strength between cohorts (all p>0.14 , Supplementary Material), indicating that the latent structure of these clinical constructs is preserved despite differences in mean levels. A RF classifier trained solely on WOMAC subscales to distinguish TKR from conservative pathways achieved an accuracy below 0.70 in both cohorts. Full details are reported in the Supplementary Material.

## 4 Discussion

This study evaluated whether stance-phase kinematic descriptors could differentiate two clinical groups of women with moderate knee osteoarthritis (KOA) sharing the same radiographic severity (KL grades 2–3) but distinct treatment pathways: scheduled total knee replacement (TKR) versus conservative management. The repeated-measures multivariate analysis identified three variables with significant Time × Treatment Group interactions surviving FDR correction: knee flexion–extension (KFE), pelvic tilt (PTILT), and back flexion–extension (SPFE). The Random Forest classifier reached a mean ROC-AUC of 0.81 using stance-phase kinematic change variables $\left(D={\text{Angle}}_{t_{3}}-{\text{Angle}}_{t_{1}}\right)$ , and six kinematic descriptors showed FDR-significant associations with joint pain. Together, these results suggest that stance-phase kinematics, derived from a standard motion capture protocol, capture functional information that is not visible from radiographic grading alone and that may contribute to the description of clinically distinct patient profiles within moderate KOA.

DPTILT emerged as the most consistent descriptor across the three analyses. It survived FDR correction in the multivariate Time × Treatment Group test, was selected by the Random Forest in 80% of cross-validation folds, and showed FDR-significant association with joint pain (AUC = 0.80) as well as a descriptive association with functional limitation (AUC = 0.73). The TKR group showed a more pronounced anterior pelvic tilt across stance, which is consistent with previous reports of compensatory trunk-pelvis postural adjustments in patients with greater knee dysfunction (Suzuki et al., 2023; Wang et al., 2016; Fu et al., 2021). Pelvic tilt during gait reflects the interaction between hip, lumbar and trunk control, and its temporal modulation across the stance phase has been associated with both pain perception and impaired motor control in KOA (Fu et al., 2021). Although our results do not establish PTILT as a stand-alone biomarker, the convergence of three different analytical approaches on this variable indicates that it captures a meaningful aspect of the functional profile that distinguishes the two groups.

The TKR group walked markedly slower than the conservative group (0.77 vs. 0.96 m/s, d = −1.02), and gait speed met both Rothman criteria for confounding of the Time × Treatment Group effect. Adjusting for velocity (Model B) attenuated the multivariate interaction (Pillai’s trace = 0.262, p = 0.125), indicating that a substantial portion of the unadjusted between-group differences in stance-phase kinematics is explained by the velocity differential. A large Time × Velocity interaction emerged (Pillai’s trace = 0.735, F = 5.040, p<0.001 , $\eta_{p}^{2}$ = 0.368), consistent with previous reports that gait speed shapes kinematic and kinetic patterns in KOA populations (Astephen et al., 2008; Mills et al., 2013). The velocity differential observed here also aligns with Tassani et al. (2022), who reported that spatiotemporal variables, including walking speed, distinguished the same two clinical groups in this cohort while kinetic variables did not. Among the three variables identified in Model A, KFE and SPFE showed strong Time × Velocity interactions and did not survive FDR adjustment in Model B, suggesting that their Time × Group effects in Model A reflect, at least in part, the slower walking pattern of the TKR group. PTILT, in contrast, retained the lowest uncorrected p -value in Model B ( p = 0.010) and did not show a significant Time × Velocity interaction ( p = 0.694), indicating that its differential change between groups is not modulated by walking speed within the stance phase.

While these results identify gait speed as a relevant factor in the multivariate analysis, its role as an individual-level descriptor for stratification is more limited. When velocity was added to the Random Forest feature pool, it was not selected in any of the cross-validation folds, and the classification performance remained essentially unchanged (AUC = 0.80 ± 0.08 vs. 0.81 ± 0.06 in the original model). This indicates that the information conveyed by walking speed is already implicitly captured by the kinematic descriptors at the individual level, particularly by those showing the strongest correlations with velocity in the present cohort (DKFE, DPOBLI, DHPFE; Supplementary Material). Velocity therefore acts as a confounder of the multivariate group comparison while being redundant for individual-level stratification, which suggests that stance-phase kinematic descriptors—and pelvic tilt in particular—carry stratification-relevant information that is not reducible to walking speed.

The three analyses, repeated-measures MANOVA, Random Forest classification, and clinical association with pain, showed partial convergence on the variables most relevant to group separation. PTILT was the only variable retained across all three approaches, and the only one whose Time × Treatment Group effect persisted at the uncorrected level after adjustment for gait speed (Model B). KFE was the strongest Time × Treatment Group effect in Model A but appeared in only 40% of RF folds and lost significance in Model B, likely reflecting that part of its stance-phase trajectory difference is shared with the velocity differential between groups, and that its net stance-phase change is less stable as an individual-level feature. SPFE showed a similar pattern. Conversely, DSHROTOFF was a stable Random Forest feature (80% of folds) and the strongest descriptor of pain (AUC = 0.81, FDR-significant), but did not show a significant Time × Treatment Group interaction in MANOVA. This pattern reflects that the three approaches evaluate different aspects of the data: MANOVA tests differences in the temporal shape of trajectories across three stance sub-phases under (un)adjusted conditions, while the Random Forest uses the net change as a single summary feature suitable for individual-level discrimination, and the clinical-association analysis links each descriptor to a specific symptomatic dimension. Treating the three approaches as complementary, rather than confirmatory, allowed identification of variables that contribute to subgroup description through different mechanisms.

Among the eight clinical outcomes evaluated, joint pain was the dimension most strongly connected to stance-phase kinematics. Six kinematic descriptors showed FDR-significant associations with pain (DSHROTOFF, DPTILT, DSPIE, DHPFE, DSPML, DSPFE), while no descriptor reached FDR significance for rigidity, depression, central sensitization, catastrophism, effusion, or synovitis. Mean cross-validated AUC across the 12 kinematic variables was higher for pain and functional limitation than for the remaining outcomes, indicating that the discriminative capacity of stance-phase kinematics is concentrated on the symptomatic dimensions most directly tied to motor function. Notably, several kinematic descriptors associated with pain showed an inverse association with depression (DPTILT, DHPFE, DSHROTOFF, DSHOBLIOFF: AUC < 0.34 for depression, AUC > 0.66 for pain). This dissociation indicates that, although pain and depression often co-occur in KOA (Sonobe et al., 2024), they may correspond to distinct stance-phase movement signatures, which has implications for how multimodal stratification frameworks combine motor and psychological dimensions.

The present results extend two previous studies from the same cohort. Tassani et al. (2022) reported that kinetic variables did not consistently differentiate treatment groups in this cohort, while spatiotemporal variables did, motivating the present focus on kinematics. Ojeda et al. (2025) characterized the same two clinical groups and found that, despite matched radiographic severity, the TKR group reported worse WOMAC, HADS, and pain catastrophizing scores than the conservative group, supporting the use of treatment pathway as a descriptor of clinical heterogeneity within moderate KOA. The present results extend these observations by showing that this clinical heterogeneity has a measurable correlate in stance-phase kinematic trajectories obtained with a standard optical motion capture protocol. Compared to kinetic measurements, kinematic descriptors require less specialized equipment, are less sensitive to force plate constraints, and could in principle be approximated with markerless or wearable systems (Uhlrich et al., 2023). This makes them a candidate component of accessible stratification frameworks, although their use in clinical decision-making would require integration with imaging and patient-reported outcomes, as recommended by OARSI/OMERACT guidelines (McAlindon et al., 2014).

Several limitations should be acknowledged. First, the analysis used $D={\text{Angle}}_{t_{3}}-{\text{Angle}}_{t_{1}}$ as a summary of stance-phase change, which does not distinguish between a difference in trajectory shape and a difference in available joint range of motion. Variables with greater intrinsic range, such as the pelvis or the trunk, may show larger D values for reasons unrelated to pathological adaptation. Continuous-curve methods such as Statistical Parametric Mapping (SPM) could in principle clarify where within the gait cycle the trajectories diverge, and could extend the analysis to the swing phase, which was not examined here and may carry complementary kinematic information; however, the most widely used implementation (SPM1d; Pataky (2012)) does not currently support multifactorial MANOVA or MANCOVA designs with continuous covariates, and its performance degrades with unbalanced group sizes. Consequently, the repeated-measures multifactorial MANCOVA framework applied here cannot be directly translated to a continuous-domain analysis without simplifying assumptions or alternative methodological frameworks beyond the scope of the present work. Second, the cohort is single-center, female-only (due to recruitment constraints), and limited to N = 66, which constrains the generalizability of the findings and reduces the statistical power of FDR correction over 12 univariate tests. Moreover, the sample size, particularly the smaller TKR subgroup (n = 27), limits the stability of feature selection and the precision of the classifier estimates. The nested cross-validation and the EPV-constrained feature selection reduce, but do not eliminate, the optimism bias inherent to small samples; the reported AUC values should therefore be interpreted with caution and validated in larger independent cohorts. Third, the Helen Hayes marker set has documented measurement limitations for transverse-plane and frontal-plane knee variables, which led us to exclude five variables *a priori*; while this decision protected the main analyses, it also limited the kinematic dimensions that could be evaluated. Fourth, the cross-sectional design does not allow inference about kinematic changes following TKR, and the comparison with the OAI cohort assessed only the consistency of the clinical constructs, not external validation of the kinematic findings. Fifth, the treatment pathway, while a useful clinical descriptor, integrates patient and clinician decisions and institutional factors that may not generalize to other settings. Sixth, the conservative group is heterogeneous by design, comprising patients managed pharmacologically, with physical therapy, or with lifestyle modifications. Although conservative interventions can alter gait kinematics (Rynne et al., 2022), the evidence needed to separate the effects of these specific therapies is not available, and the cohort was not powered to compare them; acute pharmacological effects were minimized through the washout periods described in Section 2.1. Because this within-group heterogeneity increases variance in the conservative group, it would attenuate rather than inflate the between-group differences reported here.

In summary, stance-phase kinematic trajectories differentiate two groups of women with moderate KOA who share the same radiographic severity but follow different treatment pathways. Pelvic tilt emerged as the most consistent descriptor across multivariate, individual-level, and pain-related analyses, and the most robust under adjustment for gait speed. Each analysis contributed a distinct piece of evidence: the multivariate MANOVA characterized differences in the temporal shape of stance-phase trajectories, the Random Forest evaluated individual-level discriminative capacity through summary change features, and the clinical-association analysis linked each kinematic descriptor to specific symptomatic dimensions; their partial convergence allowed identification of variables relevant to subgroup description through different mechanisms. Although the TKR group walked markedly slower than the conservative group, walking speed did not add discriminative value at the individual level beyond the kinematic descriptors, supporting kinematics as carrying stratification-relevant information not reducible to gait speed alone. Joint pain was the clinical dimension with the strongest connection to stance-phase kinematics, while psychological and inflammatory dimensions showed weaker or inverse associations. These results support the role of kinematics, obtained through standard motion capture, as an accessible alternative to kinetic measurements for describing clinical heterogeneity within moderate KOA. Validation in larger, multi-center cohorts and integration with imaging and patient-reused outcomes are necessary steps before clinical implementation.

## Data Availability

The raw data supporting the conclusions of this article will be made available by the authors, without undue reservation.

## Footnotes

1 HOLOA-DPI2016-80283-C2-1/2-R and STRATO-PID2021-126469OB-C21.
