# Supplementary material for: Stance-phase kinematic descriptors differentiate clinical subgroups in women with moderate knee osteoarthritis
Source: Front Bioeng Biotechnol. 2026 Aug 18;14:1903640. doi: 10.3389/fbioe.2026.1903640 (PMC13530001; doi:10.3389/fbioe.2026.1903640)
Supplement: Supplementary file 1 [file DataSheet1.pdf]

Supplementary Material

1 TREATMENT DECISION PROTOCOL

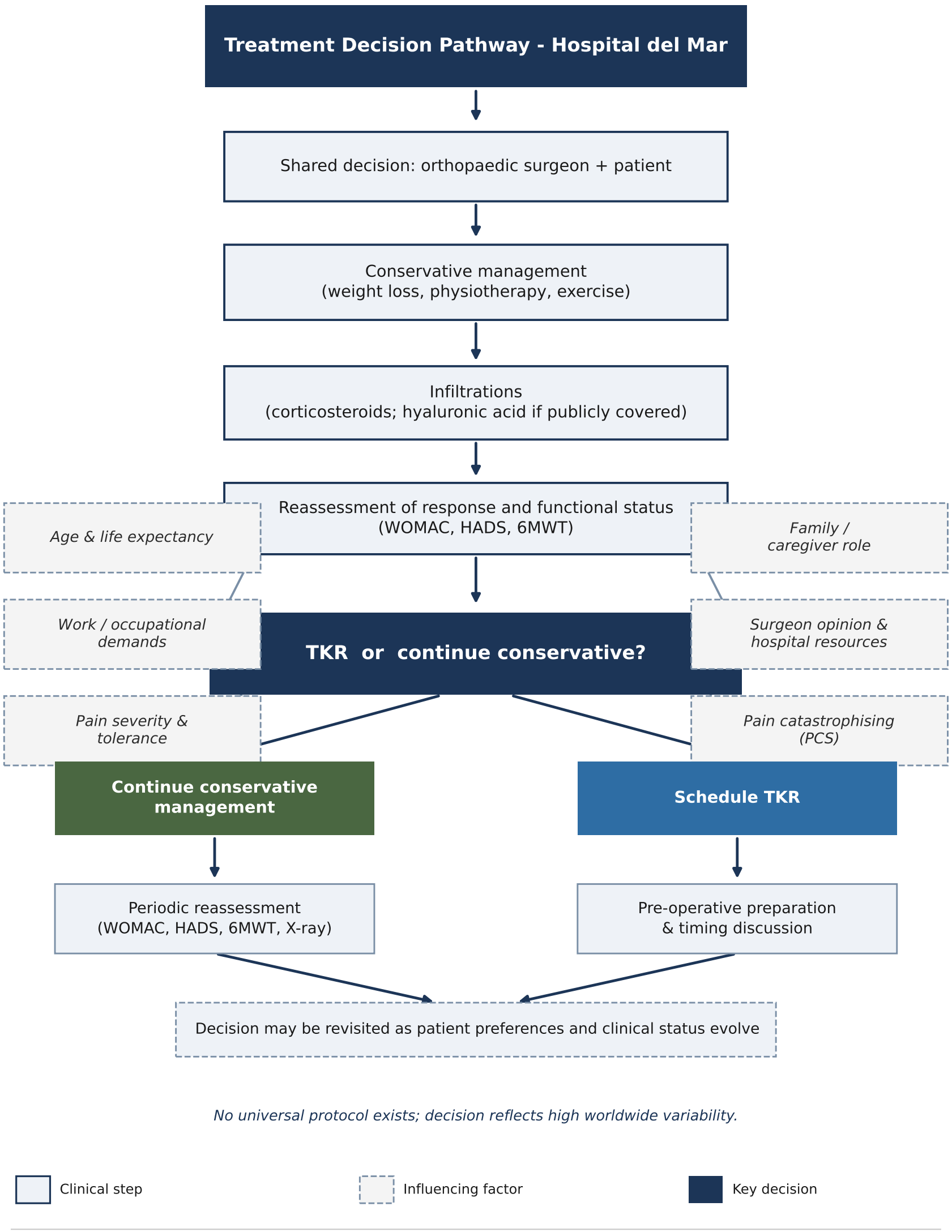

Figure S1. Treatment decision pathway at Hospital del Mar for patients with moderate knee osteoarthritis. Dashed boxes indicate patient- and context-dependent factors that influence the final decision. 6MWT, 6-Minute Walk Test; HADS, Hospital Anxiety and Depression Scale; PCS, Pain Catastrophizing Scale; TKR, total knee replacement; WOMAC, Western Ontario and McMaster Universities Osteoarthritis Index.

2 STATISTICAL ANALYSIS

2.1 Correlations between gait speed and kinematic variables

Pearson correlations between self-selected gait speed and each of the 12 retained kinematic variables were computed at the three stance sub-phases (t_1, t_2, t_3) and for the value of the angle between t_3 and t_1 (D = $Angle_{t_3} - Angle_{t_1}$). Correlations are reported for the global cohort (n = 66) and separately for the TKR (n = 27) and conservative (n = 39) subgroups. Following Rothman et al. (2008), Criterion 2 (potential confounding) is considered met when $|r| > 0.3$ and $p < .05$ in the global cohort. The full set of 36 by-timepoint correlations is reported in Tables S1–S4.

Table S1. Pearson correlations between self-selected gait speed and the value of the angle between t_3 and t_1 (D = $Angle_{t_3} - Angle_{t_1}$) of the 12 retained kinematic variables, in the global cohort and by subgroup. Variables are ordered by $|r|$ in the global cohort. Bold indicates correlations meeting Rothman's Criterion 2 in the global cohort ($|r| > 0.3$ and $p < .05$).

Variable	Global (n = 66)		TKR (n = 27)		Conservative (n = 39)		Crit. 2
	r	*p*	*r*	*p*	*r*	*p*	
**DKFE**	−**0.591**	<**0.001**	−0.737	<0.001	−0.485	0.002	Yes
**DPOBLI**	−**0.585**	<**0.001**	−0.545	0.003	−0.622	<0.001	Yes
**DAFE**	+**0.423**	<**0.001**	+0.497	0.008	+0.425	0.007	Yes
**DSPFE**	+**0.384**	**0.002**	+0.341	0.082	+0.403	0.011	Yes
**DHPFE**	−**0.367**	**0.002**	−0.878	<0.001	−0.152	0.357	Yes
**DPTILT**	−**0.324**	**0.008**	+0.017	0.932	−0.431	0.006	Yes
DSHOBLIOFF	+0.279	0.023	+0.376	0.053	+0.237	0.147	No
DSPML	+0.275	0.026	+0.508	0.007	+0.184	0.263	No
DSHROTOFF	−0.246	0.046	+0.152	0.450	−0.372	0.020	No
DSHTILTOFF	−0.124	0.320	−0.080	0.693	−0.135	0.413	No
DPROT	−0.110	0.378	−0.227	0.255	−0.118	0.476	No
DSPIE	+0.044	0.724	−0.331	0.092	+0.122	0.460	No

D = $Angle_{t_3} - Angle_{t_1}$. AFE: ankle flexion-extension; KFE: knee flexion-extension; HPFE: hip flexion-extension; PTILT: pelvic anterior-posterior tilt; POBLI: pelvic obliquity; PROT: pelvic axial rotation; SPML: back mediolateral inclination; SPIE: back internal-external rotation; SPFE: back flexion-extension; SHTILTOFF: shoulder tilt; SHOBLIOFF: shoulder obliquity; SHROTOFF: shoulder axial rotation. Six of 12 D-variables met Criterion 2 in the global cohort.

Table S2. Pearson correlations between self-selected gait speed and each kinematic variable at t_1 (loading response), in the global cohort and by subgroup. Variables are ordered by $|r|$ in the global cohort. Bold indicates correlations meeting Rothman's Criterion 2 in the global cohort ($|r| > 0.3$ and $p < .05$).

Variable at t_1	Global (n = 66)		TKR (n = 27)		Conservative (n = 39)		Crit. 2
	r	p	r	p	r	p	
**POBLI**	**+0.524**	**<0.001**	+0.562	0.002	+0.491	0.002	Yes
PTILT	−0.274	0.026	−0.088	0.664	−0.437	0.006	No
AFE	−0.270	0.028	−0.303	0.125	−0.279	0.085	No
PROT	+0.219	0.078	−0.092	0.647	+0.355	0.027	No
SPML	−0.217	0.080	−0.408	0.035	−0.109	0.511	No
KFE	+0.183	0.141	+0.276	0.164	+0.091	0.581	No
SPFE	−0.176	0.157	−0.300	0.128	−0.106	0.521	No
SHOBLIOFF	−0.151	0.227	+0.307	0.120	−0.236	0.147	No
HPFE	−0.124	0.322	+0.193	0.336	−0.256	0.117	No
SPIE	+0.064	0.607	+0.179	0.372	−0.018	0.914	No
SHTILTOFF	+0.034	0.784	+0.165	0.412	−0.098	0.553	No
SHROTOFF	−0.009	0.944	−0.072	0.722	+0.026	0.878	No

t_1: loading response. Variable abbreviations as in Table S1. One of 12 t_1 variables met Criterion 2 in the global cohort (POBLI).

Table S3. Pearson correlations between self-selected gait speed and each kinematic variable at t_2 (mid-stance), in the global cohort and by subgroup. Variables are ordered by $|r|$ in the global cohort. Bold indicates correlations meeting Rothman's Criterion 2 in the global cohort ($|r| > 0.3$ and $p < .05$).

Variable at t_2	Global (n = 66)		TKR (n = 27)		Conservative (n = 39)		Crit. 2
	r	p	r	p	r	p	
**POBLI**	**+0.341**	**0.005**	+0.350	0.073	+0.308	0.057	Yes
**HPFE**	**−0.311**	**0.011**	−0.211	0.292	−0.362	0.024	Yes
**PTILT**	**−0.302**	**0.014**	−0.029	0.886	−0.492	0.002	Yes
PROT	+0.228	0.066	−0.094	0.639	+0.377	0.018	No
SPIE	+0.152	0.223	+0.053	0.795	+0.165	0.317	No
SHOBLIOFF	−0.125	0.318	+0.373	0.056	−0.221	0.177	No
SPFE	−0.096	0.446	−0.256	0.198	−0.018	0.913	No
KFE	−0.082	0.516	−0.136	0.500	−0.096	0.563	No
SPML	−0.079	0.527	−0.103	0.609	−0.057	0.731	No
SHROTOFF	−0.072	0.564	+0.052	0.796	−0.095	0.564	No
AFE	−0.068	0.588	−0.105	0.604	−0.090	0.586	No
SHTILTOFF	+0.006	0.960	+0.136	0.498	−0.130	0.432	No

t_2: mid-stance. Variable abbreviations as in Table S1. Three of 12 t_2 variables met Criterion 2 in the global cohort (POBLI, HPFE, PTILT).

Table S4. Pearson correlations between self-selected gait speed and each kinematic variable at t_3 (terminal stance), in the global cohort and by subgroup. Variables are ordered by $|r|$ in the global cohort. Bold indicates correlations meeting Rothman's Criterion 2 in the global cohort ($|r| > 0.3$ and $p < .05$).

Variable at t_3	Global (n = 66)		TKR (n = 27)		Conservative (n = 39)		Crit. 2
	r	*p*	*r*	*p*	*r*	*p*	
**HPFE**	**−0.497**	**<0.001**	−0.586	0.001	−0.467	0.003	Yes
**PTILT**	**−0.344**	**0.005**	−0.085	0.675	−0.503	0.001	Yes
KFE	−0.262	0.033	−0.405	0.036	−0.218	0.182	No
SHROTOFF	−0.183	0.141	+0.029	0.887	−0.244	0.135	No
PROT	+0.140	0.263	−0.177	0.379	+0.272	0.094	No
AFE	+0.134	0.282	+0.028	0.888	+0.174	0.291	No
SHOBLIOFF	−0.110	0.378	+0.387	0.046	−0.203	0.216	No
SPIE	+0.094	0.453	−0.042	0.837	+0.107	0.517	No
SPFE	−0.069	0.582	−0.259	0.192	+0.014	0.933	No
SHTILTOFF	−0.016	0.896	+0.146	0.469	−0.156	0.343	No
POBLI	+0.009	0.943	+0.292	0.139	−0.142	0.389	No
SPML	−0.003	0.983	−0.028	0.890	+0.049	0.766	No

t_3: terminal stance. Variable abbreviations as in Table S1. Two of 12 t_3 variables met Criterion 2 in the global cohort (HPFE, PTILT).

2.2 Model A — main repeated-measures analysis with 12 variables

Table S5. Mauchly's sphericity test and ϵ corrections for the repeated-measures factor (Model A).

Variable	Mauchly's *W*	χ^2	df	*p*-value	GG ϵ	HF ϵ
AFE	0.463	43.884	2	<0.001	0.651	0.740
KFE	0.473	42.709	2	<0.001	0.655	0.745
HPFE	0.187	95.721	2	<0.001	0.551	0.622
PTILT	0.411	50.659	2	<0.001	0.629	0.714
POBLI	0.733	17.723	2	<0.001	0.789	0.905
PROT	0.423	48.981	2	<0.001	0.634	0.720
SPML	0.404	51.711	2	<0.001	0.626	0.711
SPIE	0.338	61.794	2	<0.001	0.602	0.682
SHTILTOFF	0.481	41.721	2	<0.001	0.658	0.749
SHOBLIOFF	0.619	27.374	2	<0.001	0.724	0.827
SHROTOFF	0.190	94.787	2	<0.001	0.552	0.623
SPFE	0.265	75.679	2	<0.001	0.576	0.652

All variables violated the sphericity assumption (all $p < 0.001$). Greenhouse-Geisser (GG) ϵ corrections were therefore applied to all univariate within-subjects tests. HF: Huynh-Feldt. The lower bound ϵ = 0.500 for all variables (df = 2).

Table S6. Univariate within-subjects results for the Time Point main effect (12-variable model, Model A). F-values and p-values were obtained with the Greenhouse-Geisser correction for sphericity violations. *Partial* η^2 represents the univariate effect size. FDR correction (Benjamini-Hochberg) was applied across all 12 variables. Statistically significant results after FDR correction ($p < 0.05$) are shown in bold.

Variable	F	p-value (GG)	p (FDR)	Partial η^2
**AFE**	**472.157**	**<0.001**	**<0.001**	**0.891**
**KFE**	**58.559**	**<0.001**	**<0.001**	**0.502**
**HPFE**	**203.951**	**<0.001**	**<0.001**	**0.779**
**POBLI**	**61.998**	**<0.001**	**<0.001**	**0.517**
**PROT**	**34.997**	**<0.001**	**<0.001**	**0.376**
**SPML**	**120.926**	**<0.001**	**<0.001**	**0.676**
**SPIE**	**53.667**	**<0.001**	**<0.001**	**0.481**
**SHTILTOFF**	**60.872**	**<0.001**	**<0.001**	**0.512**
**SHOBLIOFF**	**31.703**	**<0.001**	**<0.001**	**0.353**
**SHROTOFF**	**12.622**	**<0.001**	**<0.001**	**0.179**
SPFE	2.928	0.086	0.115	0.048
PTILT	2.528	0.108	0.130	0.042

AFE: ankle flexion-extension; KFE: knee flexion-extension; HPFE: hip flexion-extension; PTILT: pelvic anterior-posterior tilt; POBLI: pelvic obliquity; PROT: pelvic axial rotation; SPML: back mediolateral inclination; SPIE: back internal-external rotation; SHTILTOFF: shoulder tilt; SHOBLIOFF: shoulder obliquity; SHROTOFF: shoulder axial rotation; SPFE: back flexion-extension. GG: Greenhouse-Geisser. PTILT and SPFE are the only variables without a significant Time Point main effect, consistent with their role as treatment-discriminating variables through the Time × Treatment Group interaction rather than through overall temporal change.

Table S7. Univariate within-subjects results for the Time $\times$ BMI Group interaction (12-variable model, Model A). F-values and p-values were obtained with the Greenhouse-Geisser correction for sphericity violations. *Partial* η^2 represents the univariate effect size. FDR correction (Benjamini-Hochberg) was applied across all 12 variables. No variable survived FDR correction.

Variable	F	p-value (GG)	p (FDR)	Partial η^2
PTILT	6.054	0.011	0.132	0.095
POBLI	3.640	0.040	0.240	0.059
SHROTOFF	4.253	0.040	0.240	0.068
SPIE	1.559	0.219	0.876	0.026
AFE	1.389	0.250	0.876	0.023
HPFE	1.011	0.326	0.985	0.017
KFE	1.013	0.339	0.985	0.017
SHOBLIOFF	0.812	0.412	0.985	0.014
SPFE	0.550	0.485	0.985	0.009
SHTILTOFF	0.561	0.502	0.985	0.010
PROT	0.472	0.539	0.985	0.008
SPML	0.180	0.729	0.985	0.003

AFE: ankle flexion-extension; KFE: knee flexion-extension; HPFE: hip flexion-extension; PTILT: pelvic anterior-posterior tilt; POBLI: pelvic obliquity; PROT: pelvic axial rotation; SPML: back mediolateral inclination; SPIE: back internal-external rotation; SHTILTOFF: shoulder tilt; SHOBLIOFF: shoulder obliquity; SHROTOFF: shoulder axial rotation; SPFE: back flexion-extension. GG: Greenhouse-Geisser.

Table S8. Univariate within-subjects results for the Time × Treatment Group × Age Group × BMI Group interaction (12-variable model, Model A). F-values and p-values were obtained with the Greenhouse-Geisser correction for sphericity violations. *Partial* η^2 represents the univariate effect size. FDR correction (Benjamini-Hochberg) was applied across all 12 variables. No variable survived FDR correction.

Variable	F	p-value (GG)	p (FDR)	Partial η^2
SHROTOFF	5.928	0.015	0.180	0.093
SPIE	5.160	0.021	0.126	0.082
HPFE	4.320	0.038	0.152	0.069
POBLI	3.174	0.058	0.174	0.052
PTILT	3.265	0.065	0.156	0.053
KFE	2.069	0.149	0.298	0.034
SPFE	1.870	0.175	0.300	0.031
AFE	1.717	0.194	0.291	0.029
SHTILTOFF	1.440	0.240	0.320	0.024
SHOBLIOFF	1.264	0.279	0.335	0.021
PROT	0.524	0.513	0.560	0.009
SPML	0.136	0.770	0.770	0.002

AFE: ankle flexion-extension; KFE: knee flexion-extension; HPFE: hip flexion-extension; PTILT: pelvic anterior-posterior tilt; POBLI: pelvic obliquity; PROT: pelvic axial rotation; SPML: back mediolateral inclination; SPIE: back internal-external rotation; SHTILTOFF: shoulder tilt; SHOBLIOFF: shoulder obliquity; SHROTOFF: shoulder axial rotation; SPFE: back flexion-extension. GG: Greenhouse-Geisser.

Table S9. Estimated marginal means for the Time × Treatment Group interaction for kinematic variables showing significant effects in the repeated-measures MANOVA (Model A). Time points correspond to loading response (t1), mid-stance (t2), and terminal stance (t3).

Variable	Treatment Group	Time Point	Mean	SD	95% CI
**KFE**	TKR	t1	6.067	1.177	[3.710, 8.424]
		t2	4.095	1.139	[1.815, 6.375]
		t3	3.689	1.171	[1.345, 6.033]
	Conservative	t1	10.590	0.995	[8.598, 12.583]
		t2	6.632	0.963	[4.704, 8.559]
		t3	4.861	0.990	[2.879, 6.842]
**PTILT**	TKR	t1	10.937	1.260	[8.416, 13.459]
		t2	11.598	1.333	[8.930, 14.267]
		t3	12.368	1.352	[9.661, 15.074]
	Conservative	t1	8.065	1.065	[5.933, 10.197]
		t2	7.642	1.127	[5.386, 9.898]
		t3	7.559	1.143	[5.271, 9.847]
**SPFE**	TKR	t1	−16.819	2.692	[−22.209, −11.430]
		t2	−17.254	2.885	[−23.030, −11.478]
		t3	−17.303	3.012	[−23.331, −11.274]
	Conservative	t1	−18.461	2.276	[−23.017, −13.905]
		t2	−17.091	2.439	[−21.974, −12.208]
		t3	−16.401	2.546	[−21.498, −11.304]

KFE: knee flexion-extension; PTILT: pelvic anterior-posterior tilt; SPFE: back flexion-extension. CI: confidence interval. All values in degrees.

Table S10. Multivariate tests for between-subjects effects from the repeated-measures multifactorial MANOVA. *Pillai's Trace* indicates the proportion of variance explained by each effect in the multivariate model. The F statistic and corresponding p-value denote the significance of each main effect and interaction. *Partial* η^2 represents the multivariate effect size, while *Observed Power* indicates the probability of detecting a true effect given the sample size and observed variance. Statistically significant results ($p < 0.05$) are shown in bold.

Effect	Pillai's Trace	F	p-value	Partial η^2	Observed Power
Treatment Group	0.272	1.460	0.174	0.272	0.696
Age Group	0.218	1.092	0.389	0.218	0.538
BMI Group	0.330	1.933	0.054	0.330	0.839
Treatment Group × Age Group	0.136	0.616	0.818	0.136	0.298
Treatment Group × BMI Group	0.218	1.095	0.386	0.218	0.539
Age Group × BMI Group	0.093	0.401	0.956	0.093	0.194
Treatment Group × Age Group × BMI Group	0.282	1.536	0.145	0.282	0.723

All tests: F(12, 47).

2.3 Model A — repeated-measures analysis with 17 variables

Table S11. Mauchly's sphericity test and ϵ corrections for the repeated-measures factor (17-variable model, Model A).

Variable	Mauchly's *W*	χ^2	df	*p*-value	GG ϵ	HF ϵ
AFE	0.463	43.884	2	<0.001	0.651	0.740
AIE	0.124	119.085	2	<0.001	0.533	0.600
KFE	0.473	42.709	2	<0.001	0.655	0.745
KAA	0.071	150.515	2	<0.001	0.518	0.583
KIE	0.402	51.976	2	<0.001	0.626	0.710
HPFE	0.187	95.721	2	<0.001	0.551	0.622
HPAA	0.400	52.186	2	<0.001	0.625	0.709
HPIE	0.500	39.455	2	<0.001	0.667	0.759
PTILT	0.411	50.659	2	<0.001	0.629	0.714
POBLI	0.733	17.723	2	<0.001	0.789	0.905
PROT	0.423	48.981	2	<0.001	0.634	0.720
SPML	0.404	51.711	2	<0.001	0.626	0.711
SPIE	0.338	61.794	2	<0.001	0.602	0.682
SHTILTOFF	0.481	41.721	2	<0.001	0.658	0.749
SHOBLIOFF	0.619	27.374	2	<0.001	0.724	0.827
SHROTOFF	0.190	94.787	2	<0.001	0.552	0.623
SPFE	0.265	75.679	2	<0.001	0.576	0.652

All 17 variables violated the sphericity assumption (all $p < 0.001$). Greenhouse-Geisser (GG) ϵ corrections were therefore applied to all univariate within-subjects tests. HF: Huynh-Feldt. The lower bound $\epsilon = 0.500$ for all variables (df = 2). AIE: ankle internal-external rotation; KAA: knee abduction-adduction; KIE: knee internal-external rotation; HPAA: hip abduction-adduction; HPIE: hip internal-external rotation. Remaining abbreviations as in Table 3.

Table S12. Multivariate tests for within-subjects effects from the repeated-measures multifactorial MANOVA (17-variable model, Model A). Pillai's Trace is reported for each effect. The *F* statistic and corresponding *p*-value denote the significance of each effect; *Partial* η^2 represents the multivariate effect size, and *Observed Power* indicates the probability of detecting a true effect given the sample size and observed variance. Statistically significant results ($p < 0.05$) are shown in bold.

Effect	Pillai's Trace	*F*	*p*-value	Partial η^2	Observed Power
**Time Point**	**1.441**	**15.310**	**<0.001**	**0.720**	**1.000**
**Time Point × Treatment Group**	**0.514**	**2.057**	**0.001**	**0.257**	**0.999**
Time Point × Age Group	0.375	1.371	0.095	0.188	0.973
**Time Point × BMI Group**	**0.465**	**1.799**	**0.007**	**0.232**	**0.997**
Time Point × Treatment Group × Age Group	0.297	1.038	0.420	0.149	0.895
Time Point × Treatment Group × BMI Group	0.306	1.073	0.370	0.153	0.908
Time Point × Age Group × BMI Group	0.368	1.338	0.114	0.184	0.969
**Time Point × Treatment Group × Age Group × BMI Group**	**0.420**	**1.577**	**0.029**	**0.210**	**0.990**

All tests reported with hypothesis df = 34 and error df = 202.

Table S13. Univariate within-subjects results for the Time × Treatment Group interaction (17-variable model, Model A). F-values and p-values were obtained with the Greenhouse-Geisser correction for sphericity violations. *Partial* η^2 represents the univariate effect size. FDR correction (Benjamini-Hochberg) was applied across all 17 variables. Statistically significant results after FDR correction ($p < 0.05$) are shown in bold.

Variable	F	p-value (GG)	p (FDR)	Partial η^2	Observed Power
**KFE**	**9.444**	**0.001**	**0.009**	**0.140**	**0.917**
**PTILT**	**10.323**	**0.001**	**0.009**	**0.151**	**0.932**
**SPFE**	**7.966**	**0.004**	**0.023**	**0.121**	**0.832**
KAA	5.841	0.018	0.065	0.091	0.672
KIE	5.206	0.019	0.065	0.082	0.679
AFE	4.062	0.037	0.105	0.065	0.581
POBLI	2.968	0.068	0.191	0.049	0.501
SHTILTOFF	2.765	0.090	0.219	0.046	0.429
SHROTOFF	2.354	0.127	0.240	0.039	0.343
SPIE	1.290	0.268	0.456	0.022	0.217
HPIE	0.946	0.359	0.509	0.016	0.178
HPFE	1.028	0.322	0.498	0.017	0.176
SHOBLIOFF	0.488	0.555	0.709	0.008	0.116
HPAA	0.384	0.584	0.709	0.007	0.098
AIE	0.106	0.763	0.784	0.002	0.062
PROT	0.176	0.736	0.784	0.003	0.072
SPML	0.122	0.784	0.784	0.002	0.065

AFE: ankle flexion-extension; AIE: ankle internal-external rotation; KFE: knee flexion-extension; KAA: knee abduction-adduction; KIE: knee internal-external rotation; HPFE: hip flexion-extension; HPAA: hip abduction-adduction; HPIE: hip internal-external rotation; PTILT: pelvic anterior-posterior tilt; POBLI: pelvic obliquity; PROT: pelvic axial rotation; SPML: back mediolateral inclination; SPIE: back internal-external rotation; SHTILTOFF: shoulder tilt; SHOBLIOFF: shoulder obliquity; SHROTOFF: shoulder axial rotation; SPFE: back flexion-extension. GG: Greenhouse-Geisser.

Table S14. Univariate within-subjects results for the Time × BMI Group interaction (17-variable model, Model A). F-values and p-values were obtained with the Greenhouse-Geisser correction for sphericity violations. *Partial* η^2 represents the univariate effect size. FDR correction (Benjamini-Hochberg) was applied across all 17 variables. No variable survived FDR correction.

Variable	F	p-value (GG)	p (FDR)	Partial η^2
PTILT	6.054	0.011	0.187	0.095
POBLI	3.640	0.040	0.340	0.059
KIE	3.970	0.041	0.340	0.064
SHROTOFF	4.253	0.040	0.340	0.068
KAA	2.617	0.110	0.623	0.043
HPIE	1.701	0.196	0.756	0.028
SPIE	1.559	0.219	0.756	0.026
AFE	1.389	0.250	0.850	0.023
HPFE	1.011	0.326	0.850	0.017
KFE	1.013	0.339	0.850	0.017
SHOBLIOFF	0.812	0.412	0.944	0.014
SPFE	0.550	0.485	0.985	0.009
SHTILTOFF	0.561	0.502	0.985	0.010
PROT	0.472	0.539	0.985	0.008
HPAA	0.326	0.620	0.985	0.006
AIE	0.209	0.665	0.985	0.004
SPML	0.180	0.729	0.985	0.003

AFE: ankle flexion-extension; AIE: ankle internal-external rotation; KFE: knee flexion-extension; KAA: knee abduction-adduction; KIE: knee internal-external rotation; HPFE: hip flexion-extension; HPAA: hip abduction-adduction; HPIE: hip internal-external rotation; PTILT: pelvic anterior-posterior tilt; POBLI: pelvic obliquity; PROT: pelvic axial rotation; SPML: back mediolateral inclination; SPIE: back internal-external rotation; SHTILTOFF: shoulder tilt; SHOBLIOFF: shoulder obliquity; SHROTOFF: shoulder axial rotation; SPFE: back flexion-extension. GG: Greenhouse-Geisser.

3 RANDOM FOREST CLASSIFICATION WITH EXHAUSTIVE FEATURE SELECTION

3.1 Hyperparameter tuning

Table S15. Search space for randomized hyperparameter tuning of the Random Forest model. The table lists the hyperparameters considered during the tuning process, along with the set of candidate values evaluated. These parameters influence model complexity, tree structure, and ensemble behavior, and were selected to optimize model performance through randomized search.

**Hyperparameter**	**Candidate values**
Number of trees	25, 50, 100, 200, 300
Tree depth	None, 10, 20, 30, 40, 50
Minimum samples for a split	2, 5, 10
Minimum samples at leaf nodes	1, 2, 4
Number of features per split	None, `sqrt`, `log2`
Bootstrapping	True, False
Class weight	None, balanced

3.2 Classification performance metrics per cross-validation fold and overall mean. Results with maximum 5 features for EFS

Table S16. Classification performance metrics per cross-validation fold and overall mean. The table reports standard evaluation metrics including ROC-AUC, Accuracy, Precision, Recall, Specificity, F1-score, Balanced Accuracy, and the Matthews Correlation Coefficient (MCC). Values represent individual fold results and the overall mean across five outer folds. Features selected per fold via exhaustive feature selection in the inner cross-validation loop are indicated in the Features column by index: 0 = DAFE, 1 = DHPFE, 2 = DKFE, 3 = DPOBLI, 4 = DPROT, 5 = DPTILT, 6 = DSHOBLIOFF, 7 = DSHROTOFF, 8 = DSHTILTOFF, 9 = DSPFE, 10 = DSPIE, 11 = DSPML. D denotes the change of value of the angle between $t_3 - t_1$ (D = $Angle_{t_3} - Angle_{t_1}$). AFE: ankle flexion-extension; KFE: knee flexion-extension; HPFE: hip flexion-extension; PTILT: pelvic anterior-posterior tilt; POBLI: pelvic obliquity; PROT: pelvic axial rotation; SPML: back mediolateral inclination; SPIE: back internal-external rotation; SHTILTOFF: shoulder tilt; SHOBLIOFF: shoulder obliquity; SHROTOFF: shoulder axial rotation; SPFE: back flexion-extension.

**Fold**	**Features**	**AUC**	**Accuracy**	**Precision**	**Recall**	**Specificity**	**F1**	**Bal. Acc.**	**MCC**
1	0, 2, 4, 10	0.771	0.714	0.750	0.500	0.875	0.600	0.688	0.411
2	1, 5, 7, 10	0.800	0.692	0.600	0.600	0.750	0.600	0.675	0.350
3	2, 4, 5, 7, 10	0.925	0.846	0.714	1.000	0.750	0.833	0.875	0.732
4	1, 4, 5, 7, 10	0.800	0.692	0.667	0.400	0.875	0.500	0.638	0.318
5	1, 5, 7, 8, 9	0.762	0.615	0.571	0.667	0.571	0.615	0.619	0.238
**Mean**	—	**0.812**	**0.712**	**0.660**	**0.633**	**0.764**	**0.630**	**0.699**	**0.410**

Bal. Acc.: balanced accuracy; MCC: Matthews correlation coefficient.

3.3 Results with maximum 3 features for EFS

Table S17. Classification performance metrics per cross-validation fold and overall mean for the Random Forest model with a maximum of three features per fold (EFS, 5×5 nested cross-validation). Features selected per fold via exhaustive feature selection in the inner cross-validation loop are indicated in the Features column by index: 0 = DAFE, 1 = DHPFE, 2 = DKFE, 3 = DPOBLI, 4 = DPROT, 5 = DPTILT, 6 = DSHOBLIOFF, 7 = DSHROTOFF, 8 = DSHTILTOFF, 9 = DSPFE, 10 = DSPIE, 11 = DSPML. D denotes the change of value of the angle between $t_3 - t_1$ (D = $Angle_{t_3} - Angle_{t_1}$). AFE: ankle flexion-extension; KFE: knee flexion-extension; HPFE: hip flexion-extension; PTILT: pelvic anterior-posterior tilt; POBLI: pelvic obliquity; PROT: pelvic axial rotation; SPML: back mediolateral inclination; SPIE: back internal-external rotation; SHTILTOFF: shoulder tilt; SHOBLIOFF: shoulder obliquity; SHROTOFF: shoulder axial rotation; SPFE: back flexion-extension.

Fold	Features	AUC	Accuracy	Precision	Recall	Specificity	F1	Bal. Acc.	MCC
1	0, 2, 4	0.812	0.714	0.750	0.500	0.875	0.600	0.688	0.411
2	2, 4, 10	0.600	0.538	0.444	0.800	0.375	0.571	0.588	0.184
3	1, 5, 10	0.950	0.923	0.833	1.000	0.875	0.909	0.938	0.854
4	1, 4, 5	0.725	0.615	0.500	0.400	0.750	0.444	0.575	0.158
5	0, 1, 5	0.738	0.615	0.571	0.667	0.571	0.615	0.619	0.238
**Mean**	—	**0.765**	**0.681**	**0.620**	**0.673**	**0.689**	**0.628**	**0.681**	**0.369**

Bal. Acc.: balanced accuracy; MCC: Matthews correlation coefficient. EFS: exhaustive feature selection.

3.4 Random Forest classification with 12 kinematic variables and mean velocity as features

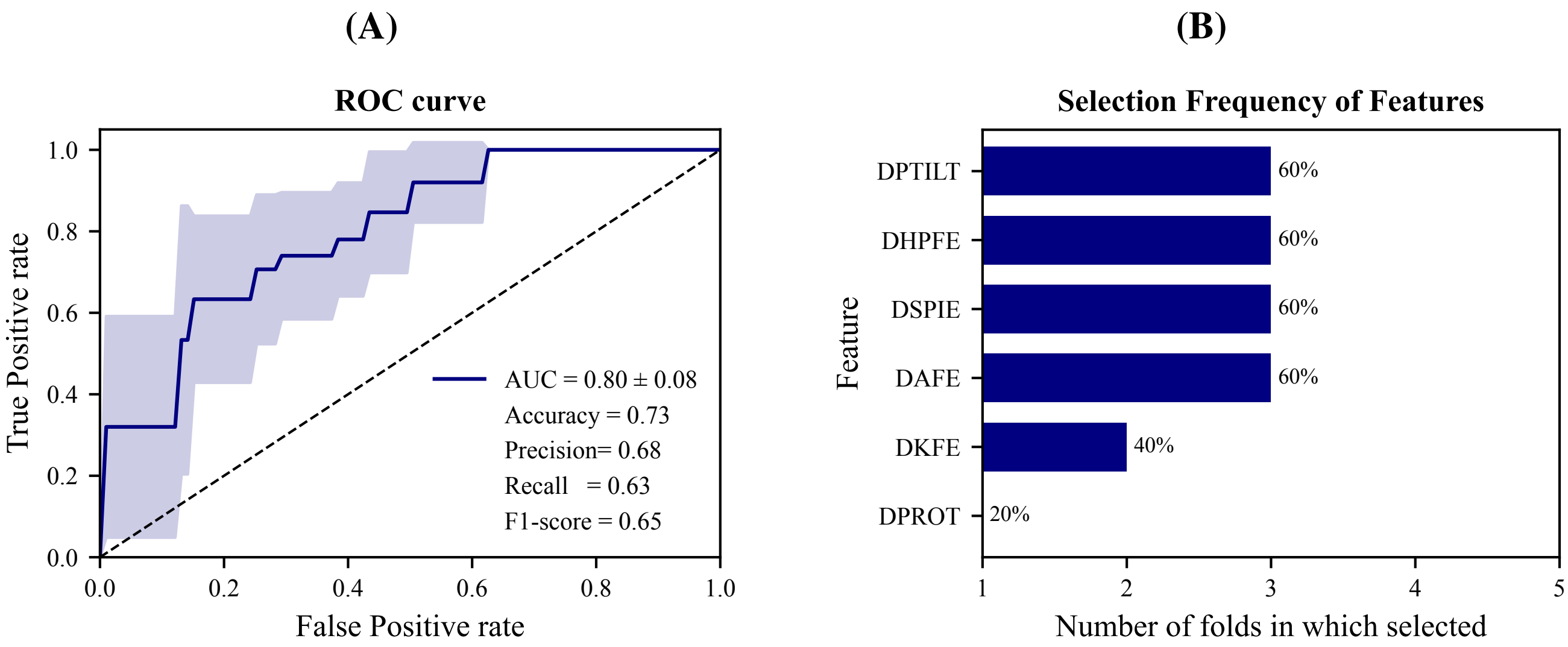

Figure S2. Results from nested cross-validation with exhaustive feature selection (EFS) using a Random Forest classifier trained on 12 kinematic change scores and mean gait velocity. (**A**) Mean Receiver Operating Characteristic (ROC) curve across five outer folds. The blue line represents the mean ROC curve, the shaded area indicates ± 1 standard deviation of the true positive rate, and the dashed diagonal line corresponds to random classification (AUC = 0.80 $\pm$ 0.08). (**B**) Frequency of feature selection across outer folds. Each bar represents the number of folds (out of five) in which a given feature was selected as part of the optimal subset. Features appearing in multiple folds indicate higher selection stability.

4 CLINICAL CONSTRUCT CONSISTENCY AGAINST THE OAI COHORT

4.1 Normality and homogeneity assessments

Table S18. Normality (Shapiro–Wilk) and homogeneity of variances (Levene's test) for WOMAC outcome variables in the HOLOA/STRATO and OAI cohorts. Values reported are *p*-values.

Variable	HOLOA/STRATO Shapiro–Wilk *p*	OAI Shapiro–Wilk *p*	Levene's test *p*-value
Pain	0.220	<0.001	0.008
Rigidity	0.013	<0.001	<0.001
Functionality	0.306	<0.001	<0.001

WOMAC: Western Ontario and McMaster Universities Osteoarthritis Index. OAI: Osteoarthritis Initiative. Shapiro–Wilk tests normality within each cohort; Levene's test assesses homogeneity of variances between cohorts. Violations of normality and homoscedasticity in the OAI cohort justified the use of Mann–Whitney *U* tests for between-cohort comparisons.

Table S19. Fisher *r*-to-*z* comparison of WOMAC subscale correlations between the HOLOA/STRATO and OAI cohorts.

Constructs	$r_{\text{HOLOA/STRATO}}$	r_{OAI}	z	*p*-value
Pain–Rigidity	0.531	0.657	−1.473	0.141
Pain–Functionality	0.835	0.848	−0.321	0.748
Rigidity–Functionality	0.616	0.713	−1.310	0.190

No significant differences in correlation strength were found between cohorts (all $p > 0.14$), indicating that the latent structure of WOMAC subscales is preserved across the HOLOA/STRATO and OAI datasets.

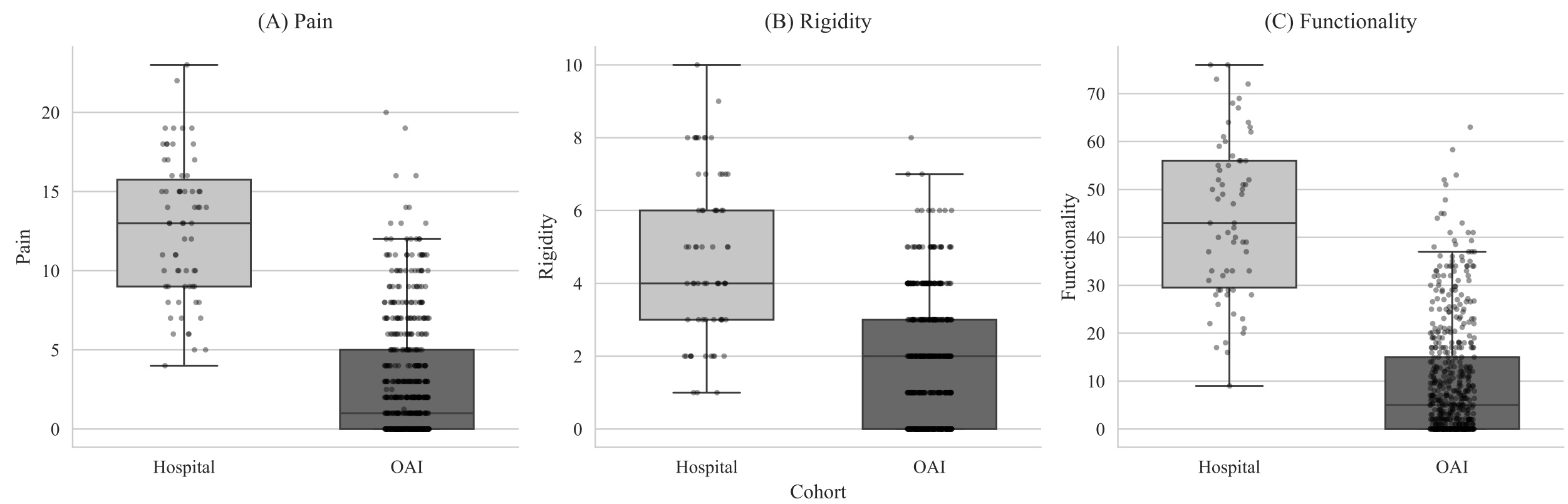

Figure S3. Distribution of WOMAC subscale scores across cohorts. Raincloud plots showing the distribution of Pain (A), Rigidity (B), and Functionality (C) scores from the Western Ontario and McMaster Universities Osteoarthritis Index (WOMAC) in the Hospital (n = 66) and OAI (n = 447) cohorts. Each panel combines three layers: a half-violin (kernel density estimate, KDE) reflecting the shape of the distribution, a box plot indicating the median and interquartile range (IQR) with whiskers extending to 1.5 × IQR, and individual data points. Between-group differences were assessed using the two-sided Mann–Whitney U test. Significance levels: * p < 0.05; ** p < 0.01; *** p < 0.001; ns, not significant.

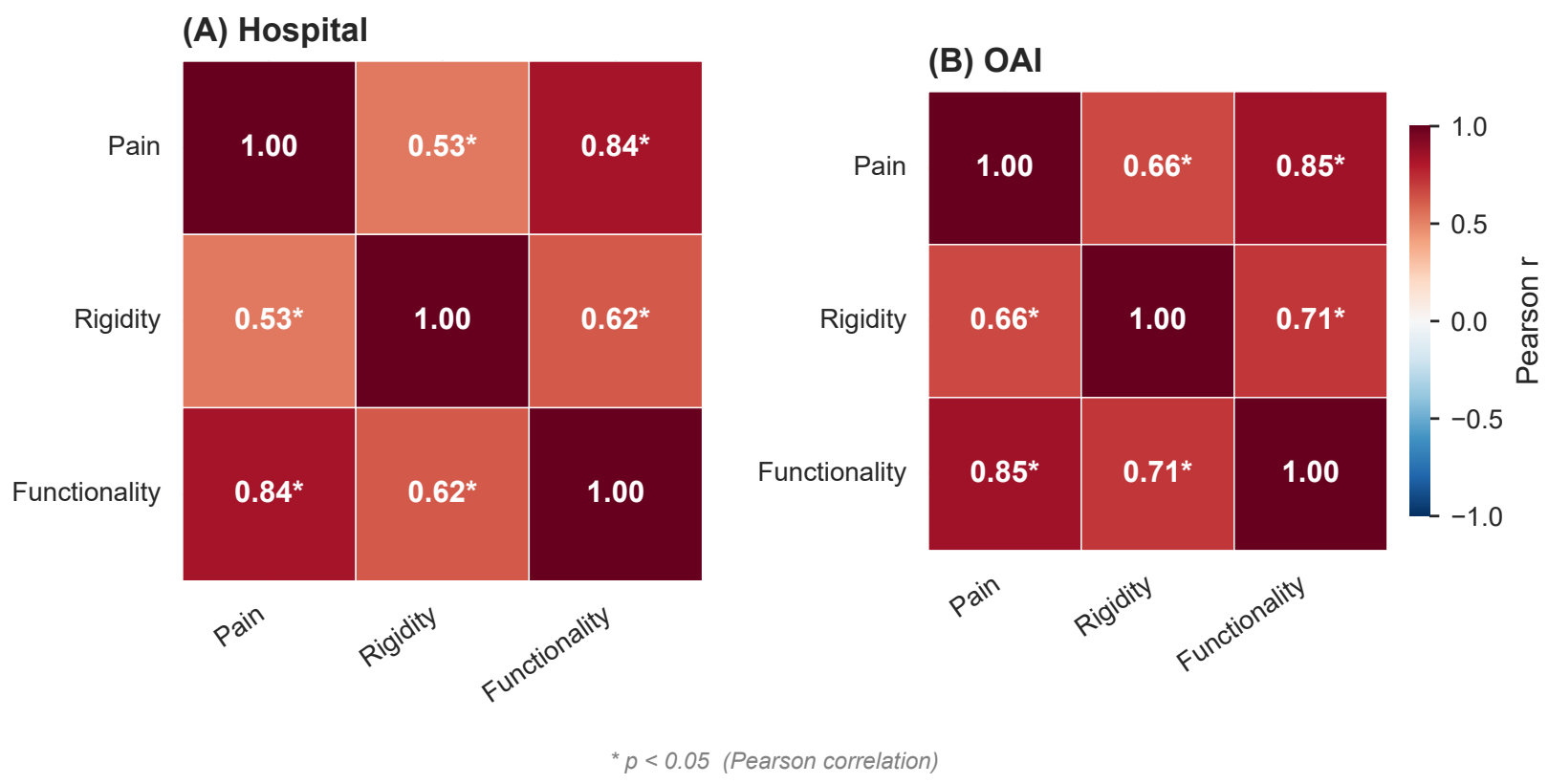

Figure S4. Heatmaps of Pearson correlations between pain, rigidity, functionality for HOLOA/STRATO and OAI cohort

4.2 Principal Component Comparison

Principal Component Analysis (PCA) was performed separately on the Hospital del Mar (HOLOA/STRATO) and OAI datasets using the same WOMAC variables (functionality, pain, and rigidity). For each dataset, the explained variance ratio was computed, and the cumulative explained variance was used to identify the optimal number of components, with a threshold of 0.80. We extracted the loadings of the first and second principal components (PC1 and PC2) from HOLOA/STRATO and OAI and compared them to assess similarity in the contributions of the WOMAC variables. Loadings represent the contribution of each variable to the component and are indicative of the direction and strength of each feature's influence.

Cosine Similarity Evaluation

To quantify the similarity between PC1 vectors from the two datasets, cosine similarity was calculated:

$$\text{Cosine Similarity} = \frac{\mathbf{u} \cdot \mathbf{v}}{\|\mathbf{u}\| \|\mathbf{v}\|}$$

where $\mathbf{u}$ and $\mathbf{v}$ are the PC1 loading vectors from the original (HOLOA/STRATO) and external (OAI) datasets, respectively, $\mathbf{u} \cdot \mathbf{v}$ is their dot product, and $\| \cdot \|$ denotes the Euclidean norm. A cosine similarity score close to 1 indicates high directional similarity, supporting the reproducibility of biomechanical features across the two patient populations.

In the OAI dataset, a single principal component explained over 80% of the variance, whereas in the HOLOA/STRATO dataset, two components were required to reach a level of explained variance over 80%. In the HOLOA/STRATO dataset, PC1 explained 77.7% of the variance, and PC2 explained 17.1%, with both components together capturing 94.8% of the total variance. In the OAI dataset, the first two components explained 95.1% of the variance; PC1 (82.8%) alone exceeded the cumulative threshold and PC2 accounted for 12.3% of the variance. The loadings of PC1 and PC2 for the WOMAC subscales (Table S20) were examined to compare the PCA structures across datasets. In both datasets, the loadings were similar, suggesting a comparable underlying structure. Given that PC1 accounted for the majority of variance in both datasets, cosine similarity analyses focused on PC1, obtaining a high value of 0.99

Table S20. Principal component analysis (PCA) loadings for WOMAC subscales in the OAI and HOLOA/STRATO cohorts. PC1 explained 82.8% and 77.7% of the variance in the OAI and HOLOA/STRATO datasets, respectively; PC2 explained 12.3% and 17.1%, with both components jointly accounting for over 94% of total variance in each cohort.

**Subscale**	**OAI**	**HOLOA/STRATO**
**PC1**		
Functionality	0.60	0.61
Pain	0.58	0.59
Rigidity	0.55	0.52
**PC2**		
Functionality	−0.22	−0.26
Pain	−0.54	−0.47
Rigidity	0.81	0.84

PC: principal component; WOMAC: Western Ontario and McMaster Universities Osteoarthritis Index. Values represent component loadings. OAI: Osteoarthritis Initiative.

between the PC1 loading vectors.

4.3 Classification Analysis Based on WOMAC Variables with Random Forest and SVM

To explore the discriminative capacity of baseline WOMAC subscale scores in classifying patients undergoing knee arthroplasty versus those treated conservatively, the same pipeline used with the biomechanical variables was implemented using the HOLOA/STRATO cohort: a random forest model with randomized search and exhaustive feature selection.

The data was split into training, test and validation sets using a 60/20/20 stratified partition. The model's performance was evaluated on the test set across the following metrics: accuracy, precision, recall, F1-score and ROC-AUC. The accuracy (0.64), the precision (1.00), recall (0.00), and F1-score (0.00) revealed that the classification power of these clinical variables is weak, suggesting that they do not provide strong predictive information regarding the treatment group. The dataset is balanced, thus ruling out the possibility that the error is due to class imbalance. These metrics reinforce the notion that WOMAC subscale scores alone lack sufficient discriminative power to reliably differentiate between surgical and conservative treatment groups. Taken together, these findings highlight the need for incorporating additional clinical, imaging, or patient-reported outcome variables to enhance predictive performance.

A similar classification attempt was conducted on the OAI dataset using WOMAC subscales as input features and a Random Forest classifier. However, the OAI dataset was substantially imbalanced with respect to treatment assignment, and even after applying class weights, classification performance remained poor. To address this, an alternative supervised classifier, a Support Vector Machine (SVM), was explored. The OAI dataset was divided according to the treatment type indicator variable `P02KRS3`, where 0 denotes conservative treatment and 1 denotes TKR. Due to class imbalance, with a larger number of patients in the conservative group, a random undersampling strategy was employed. Specifically, in each of 100 independent iterations, a random sample of 14 patients was drawn from the conservative treatment

Table S21. Classification performance of a Random Forest model trained exclusively on WOMAC subscales to predict treatment group (TKR vs. conservative). Results are reported for the OAI and HOLOA/STRATO cohorts.

Dataset	AUC	Accuracy	Precision	Recall	F1-score
OAI (balanced, 100 iterations)	0.72	0.68	0.54	0.52	0.49
HOLOA/STRATO (balanced, full sample)	0.29	0.40	0.33	0.50	0.40

WOMAC: Western Ontario and McMaster Universities Osteoarthritis Index. AUC: area under the ROC curve. Balanced sampling was applied to account for class imbalance between treatment groups.

group (without replacement), and this subset was combined with the full arthroplasty group to construct a balanced dataset for classification. For each iteration, the classification model was trained using the following baseline WOMAC subscale variables as predictors:

- `V00WOMADLR` – WOMAC pain score
- `V00WOMKPR` – WOMAC physical function score
- `V00WOMSTFR` – WOMAC stiffness score

The data were split into training and test sets using an 80/20 stratified partition. An SVM classifier with a linear kernel and probabilistic output was fitted to the training data. The model's performance was evaluated on the test set across the following metrics: accuracy, precision, recall, F1-score and ROC-AUC. ROC- AUC was computed only when both classes were present in the test split. In cases where only one class was present, ROC AUC was not computed, and a counter was used to record such instances. In total, 100 iterations were conducted. The mean performance metrics across iterations are depicted in Table S21. Although the mean ROC AUC across the 100 balanced iterations reached 0.72—indicating some degree of ability to rank patients by likelihood of surgical intervention—the overall classification performance was suboptimal. The mean accuracy was 0.68, but the precision (0.54), recall (0.52), and F1-score (0.49) revealed limited sensitivity and poor class balance. Moreover, in 7 out of the 100 iterations, the ROC AUC could not be computed due to the absence of class variation in the test subset. These findings underscore the limitations of relying solely on WOMAC subscales for treatment classification.

To further examine this observation, a similar SVM classification approach was applied to the HOLOA/STRATO cohort dataset using the same WOMAC subscales as input features. In this case, without subsampling, the model yielded considerably poorer results: an accuracy of 0.40, precision of 0.33, recall of 0.50, F1-score of 0.40, and ROC AUC of 0.29. These metrics collectively underscore the limited discriminative power of baseline WOMAC subscale scores for distinguishing between patients managed conservatively and those scheduled for arthroplasty. Taken together, these findings highlight the need for incorporating additional clinical, imaging, or patient-reported outcome variables to enhance predictive performance.

REFERENCES

Rothman, K. J., Greenland, S., and Lash, T. L. (2008). *Modern Epidemiology* (Philadelphia: Lippincott Williams Wilkins), 3rd edn.
